# Toxicity and Growth Assessments of Three Thermophilic Benthic Dinoflagellates (*Ostreopsis* cf. *ovata, Prorocentrum lima* and *Coolia monotis*) Developing in the Southern Mediterranean Basin

**DOI:** 10.3390/toxins8100297

**Published:** 2016-10-15

**Authors:** Hela Ben-Gharbia, Ons Kéfi-Daly Yahia, Zouher Amzil, Nicolas Chomérat, Eric Abadie, Estelle Masseret, Manoella Sibat, Habiba Zmerli Triki, Habiba Nouri, Mohamed Laabir

**Affiliations:** 1 Research Group on Oceanography and Plankton Ecology, Tunisian National Institute of Agronomy (INAT), IRESA—Carthage University, 43 Avenue Charles Nicolle, Tunis 1082, Tunisia; onsdaly@yahoo.fr (O.K.-D.Y.); bibarouma@hotmail.fr (H.Z.T.); 2 Phycotoxins Laboratory, Institut Français de Recherche pour l’Exploitation de la Mer (IFREMER), Rue De l’Ile d’Yeu, BP 21105, Nantes Cedex 3 F-44311, France; zouher.amzil@ifremer.fr (Z.A.); manoella.sibat@ifremer.fr (M.S.); 3 Laboratoire Environnement Ressource de Bretagne Occidentale (LER-BO), Institut Français de Recherche pour l’Exploitation de la Mer (IFREMER), Station de Biologie Marine, Place de la Croix, BP 40537, Concarneau F-29185, France; Nicolas.Chomerat@ifremer.fr; 4 Center for Marine Biodiversity, Exploitation and Conservation (MARBEC), Montpellier University, Centre National de la Recherche Scientifique (CNRS), Institut de Recherche pour le Développement (IRD), Institut Français de Recherche pour l’Exploitation de la Mer (IFREMER), Place Eugène Bataillon, CC093, Montpellier Cedex 5 F-34095, France; eric.abadie@ifremer.fr (E.A.); estelle.masseret@univ-montp2.fr (E.M.); mohamed.laabir@univ-montp2.fr (M.L.); 5 Institut de Recherche pour le Développement (IRD), 2 Rue Des Sports-El Menzah 1, BP 434, Tunis 1004, Tunisie; habiba.nouri@ird.fr

**Keywords:** *Ostreopsis* cf. *ovata*, *Prorocentrum lima*, *Coolia monotis*, Southern Mediterranean Sea, growth, toxicity

## Abstract

Harmful benthic dinoflagellates, usually developing in tropical areas, are expanding to temperate ecosystems facing water warming. Reports on harmful benthic species are particularly scarce in the Southern Mediterranean Sea. For the first time, three thermophilic benthic dinoflagellates (*Ostreopsis* cf. *ovata*, *Prorocentrum lima* and *Coolia monotis*) were isolated from Bizerte Bay (Tunisia, Mediterranean) and monoclonal cultures established. The ribotyping confirmed the morphological identification of the three species. Maximum growth rates were 0.59 ± 0.08 d^−1^ for *O.* cf. *ovata*, 0.35 ± 0.01 d^−1^ for *C. monotis* and 0.33 ± 0.04 d^−1^ for *P. lima.* Toxin analyses revealed the presence of ovatoxin-a and ovatoxin-b in *O.* cf. *ovata* cells. Okadaic acid and dinophysistoxin-1 were detected in *P. lima* cultures. For *C. monotis*, a chromatographic peak at 5.6 min with a mass *m*/*z* = 1061.768 was observed, but did not correspond to a mono-sulfated analogue of the yessotoxin. A comparison of the toxicity and growth characteristics of these dinoflagellates, distributed worldwide, is proposed.

## 1. Introduction

Harmful algal blooms (HABs) seem to have become more frequent, intense and widespread [1]. These events occur preferentially in coastal waters and sheltered areas throughout the world, such as harbors, small bays and coastal lagoons [2]. This phenomenon has been attributed either to global climate changes, to anthropogenic impacts or to the worldwide increase in monitoring programs [3]. Toxic and non-toxic HABs species are usually associated with disastrous effects on ecosystems, human health and on economic activities [1,4,5].

Great attention has been given to planktonic taxa responsible for HAB outbreaks; however, marine epiphytic dinoflagellate communities are now attracting increasing interest due to the expansion of their biogeographic areas (from tropical-subtropical to temperate waters), showing a more frequent occurrence at higher latitudes [6,7]. The presence of new thermophilic harmful species in the Mediterranean basin has been documented in the Northern Mediterranean Sea. Their occurrence has probably been promoted by the increase of water temperature during the last few decades [8,9]; as this factor represents one of the main environmental drivers affecting growth and bloom development of phytoplanktonic species [1,10,11]. *Ostreopsis ovata*, *Prorocentrum lima* and *Coolia monotis* often constitute a significant part of potentially toxic epiphytic dinoflagellate assemblages. No correlation (competition and/or facilitation) between the abundance of the three taxa was highlighted [12]. *O. ovata* was taxonomically identified by Fukuyo (1981) [13] from coral reefs of French Polynesia. This species represented a major cause of toxic blooms in the Northern Mediterranean Sea [14,15] and was associated with human diseases [16,17]. The most extensive sanitary events occurred in Italy (2005–2006), Spain (2004), Algeria (2009) and France (2006–2009) [18]. *O. ovata* can form floating clusters at the seawater surface and releases marine aerosols, causing thereby respiratory problems and irritations [19]. It can produce palytoxin (PLTX) [20,21,22,23], ovatoxins (OVTXs) [24,25,26,27] and mascarenotoxins [28]. A regulatory level of 30 µg of the sum of palytoxins and ostreocin-d per kg of body tissues has been proposed for the harvesting and consumption of shellfish resources by the European Food Safety Authority [28].

*P. lima* was initially described in the Mediterranean Sea, in the Gulf of Sorrento in Italy [29]. This species is abundant, cosmopolitan and distributed worldwide. It is known to produce several toxic molecules, such as okadaic acid (OA) and its analogues [30,31,32,33,34,35,36], dinophysistoxins (DTXs) [32,37,38,39,40], prorocentrolide [41] and prorocentin [42]. *P. lima* has been associated with diarrhetic shellfish poisoning (DSP) in different parts of the world [43,44,45,46,47] and has been suspected to contribute to the Ciguatera syndrome [48]. A maximum limit of 160 µg for combined okadaic acid, dinophysistoxins and pectenotoxins per kg of edible tissues was established by the EFSA (European Food Safety Authority) [49].

Concerning *C. monotis*, which was firstly described by Meunier (1919) [50] in oyster parks of North European waters (North Sea: Deswartes, Nieuport, Belgium), there has been some confusion about its taxonomy and toxicity [51]. *C. monotis* seems to be non-toxic or to include toxic and non-toxic strains. It was previously thought to produce cooliatoxin [52], to have hemolytic activity [53,54] and to be toxic to mice [52,55] and to *Artemia salina* and *Haliotis virginea* larvae [56]. However, the classification of these toxic strains was revised, and they were re-identified as *Coolia tropicalis* or *Coolia malayensis* [57]. Until now, no blooms or human health problems have been associated with *C. monotis.*

Recent works allowed a better understanding of the taxonomy, phylogeny, toxicity and autoecology of these three potentially toxic benthic dinoflagellates [3,15,23,58,59,60,61,62,63,64,65]. Nevertheless, more research on toxic strains is needed, particularly in the Southern Mediterranean basin. Reports about the occurrence of these species along the southern coasts are limited to Tunisia [66,67,68,69,70,71,72,73], Egypt [74,75] and Algeria [76]. These studies were based only on fixed field samples. No cultures were established, and no molecular characterizations were performed. Data on the toxicity of these southern Mediterranean species do not exist.

Our work aims to fully identify *O. ovata*, *P. lima* and *C. monotis* from a southern Mediterranean ecosystem and to gain more insight concerning their physiology (growth and toxin production). Genetic characterization of these three benthic dinoflagellates was performed to establish the phylogenetic relationship with other globally-distributed strains. The toxin profiles and contents of the three benthic dinoflagellates were determined using the liquid chromatography mass spectrometry technique. Data on the toxic and growth characteristics of the three dinoflagellates were compared to previously-reported ones, from a large spectrum of marine ecosystems.

## 2. Results and Discussion

### 2.1. Culture Observations

Live cells from the three strains were characterized by a distinctive behavior. *O*. cf. *ovata* cells swam with a geotropic orientation as previously described by Aligizaki and Nikolaidis (2006) [77]. Cells tended to be attached to flask walls and to be embedded in mucous, forming a brownish spider web. This species displayed a high morphological variability with the presence of small percentages of small/dark cells and thin-/double-walled cysts as reported by Accoroni et al. (2014) [78]. *P. lima* cells were attached to the bottom of the flasks forming dense mats. Cells formed aggregates and were motionless to weakly motile as described by Marr et al. (1992) [32]. *C. monotis* cells were very active and showed a rotational movement in a clockwise direction. Cells produced mucus, and many brownish lines were observed in the culture medium. Many benthic dinoflagellates, such as *Ostreopsis* spp., *Prorocentrum* spp., *Coolia* spp. and *Gambierdiscus toxicus*, are known to produce copious amounts of mucilage both in situ and in culture [23,79]. This feature can offer competitive advantages to these species by facilitating their attachment to surfaces, leading to the colonization of different substrates. Mucilage seems also to act as a defense against grazing, to be involved in metabolic self-regulation and to play a key role in reducing dispersion, increasing buoyancy and conveying toxicity [80,81,82].

### 2.2. Morphology

#### 2.2.1. *Ostreopsis* cf. *ovata*

**Figure 1:**
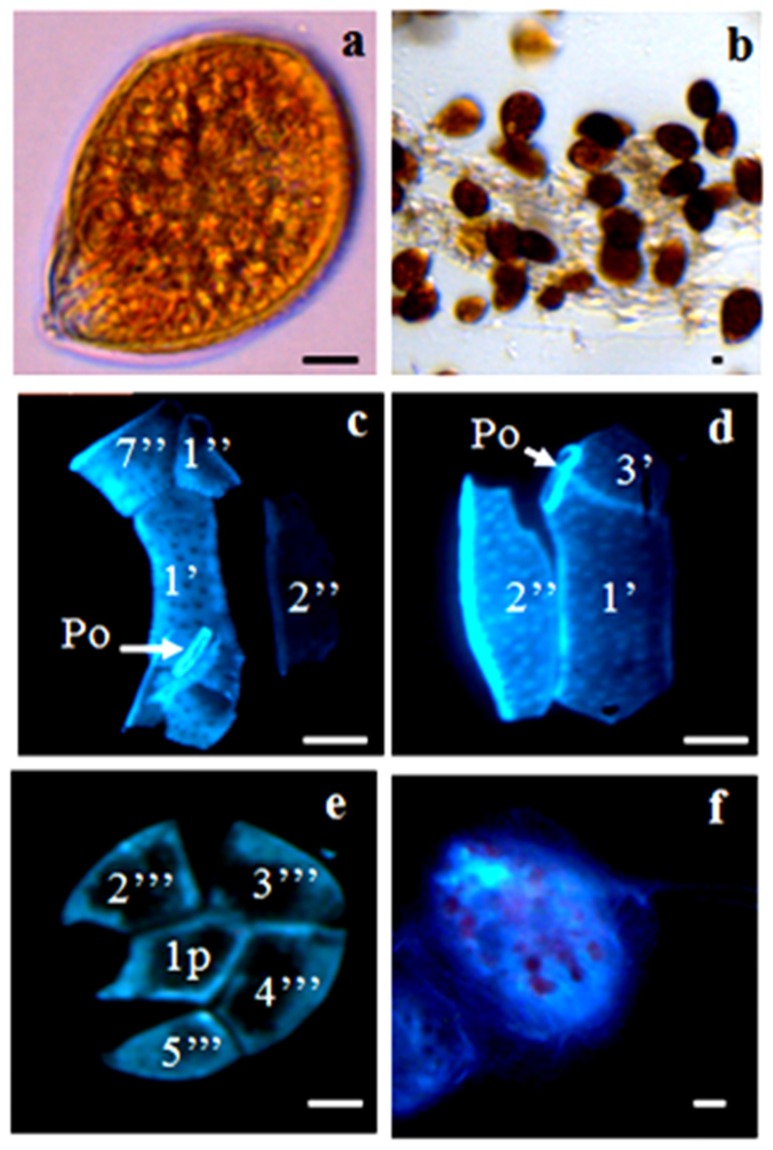
Vegetative cells of *Ostreopsis* cf. *ovata*, observed under light microscopy (**a**,**b**) and after calcofluor (**c**–**e**) and DAPI (4’,6-diamidino-2-phenylindole dihydrochloride) staining (**f**): (**a**) single cell; (**b**) cells embedded in mucous; (**c,d**) epithecal view; (**e**) hypothecal view. Scale bars, 10 µm. Po: pore plate.

Vegetative cells were ovoid to oblong, pointed toward the ventral area in apical view, with many golden chloroplasts. A sub-spherical nucleus was located at the posterior end of the cell (Figure 1). For cells harvested in the exponential growth phase, mean length and width were 50.38 ± 4.36 µm and 36.80 ± 3.33 µm, respectively. Vegetative cell sizes of the *O*. cf. *ovata* OOBZT14 strain matched those reported for the Mediterranean populations and were close to those found by other authors [26,74,78,83]. During the stationary growth phase, cells with more rounded and irregular shapes were observed in *O.* cf. *ovata* cultures with a mean length and width of 55.18 ± 5.25 µm and 39.58 ± 3.61 µm, respectively (Table 1). Our results are in agreement with those of Accoroni et al. (2012) [84], Vanucci et al. (2012) [85] and Pezzolezi et al. (2014) [86], who reported an increase in cell size and biovolume at the stationary and the decline phases of the cultures. The appearance of these large anomalous cells can be interpreted as a response to unfavorable conditions (nutrient depletion) or as a precursor of pellicle cysts at the end of the growth phase [83].

#### 2.2.2. *Prorocentrum lima*

**Figure 2:**
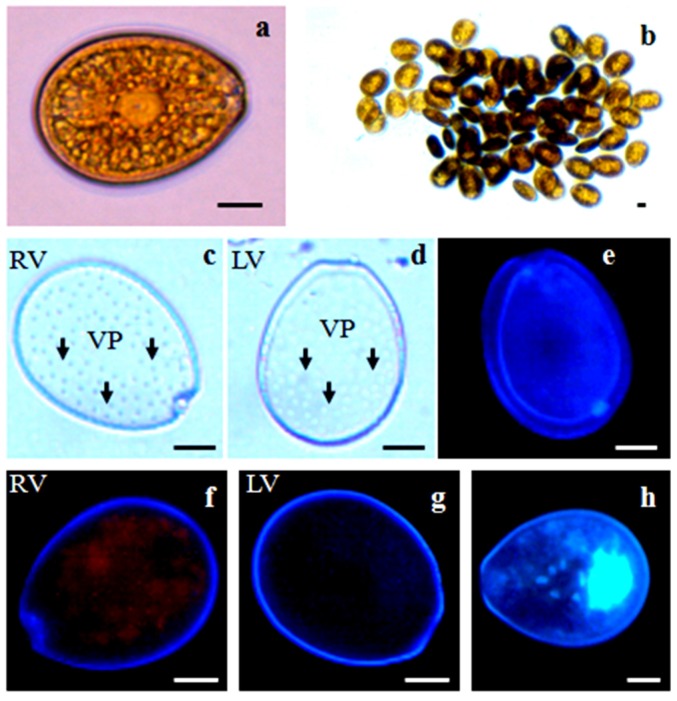
Vegetative cells of *Prorocentrum lima*, observed under light microscopy (**a**–**d**) and after calcofluor (**e**–**g**) and DAPI staining (**h**): (**a**) single cell; (**b**) cell aggregate; (**c,f**) V-shaped right valves; (**d,g**) left valves; (**h**) nucleus located at the dorsal part of the cell. Scale bars, 10 µm. RV: right valve; LV: left valve; VP: valve pores.

Cells were oval to oblong in valve view, and both valves were concave in lateral view. The periflagellar area was V-shaped and located on the right valve. The ring-shaped pyrenoid was situated in the center of the cell, and the nucleus occupied the dorsal part (Figure 2). Cell length varied from 42.98–48.80 µm and width 34.941–37.95 µm. The variation in cell shape, expressed by the length/width ratio, ranged from 1.23–1.31. No size differences (*p* > 0.05) were observed between cells in exponential and stationary growth phases (Table 1). Cell sizes of *P. lima* from Tunisian waters fit well with the description of Aligizaki et al. (2009) [87] and Aissaoui et al. (2014) [70].

#### 2.2.3. *Coolia monotis*

**Figure 3:**
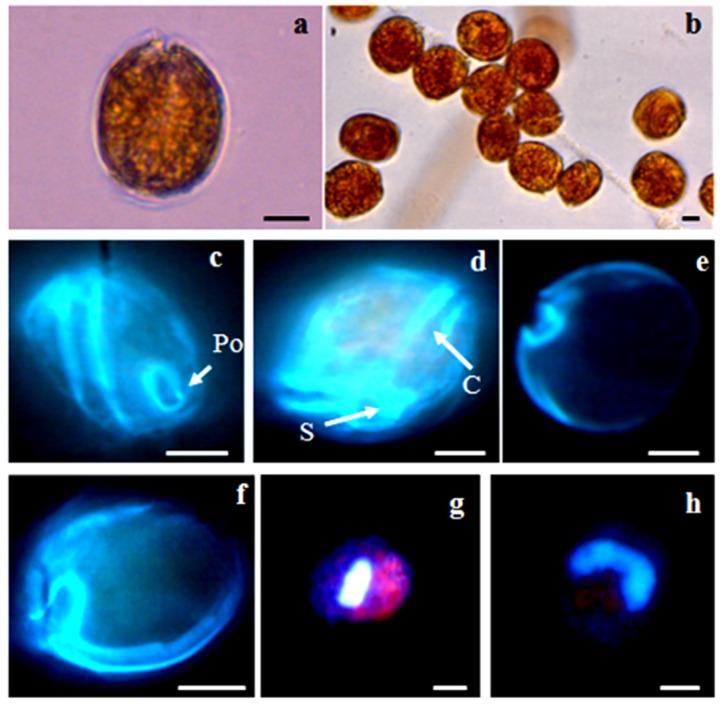
Vegetative cells of *Coolia monotis*, observed under light microscopy (**a**,**b**) and after calcofluor (**c**–**f**) and DAPI staining (**g**,**h**): (**a**) single cell; (**b**) cells embedded in mucous; (**c**) side view; (**d**) ventral view; (**e**,**f**) hypothecal view (**g**,**h**) U-shaped nucleus located in the dorsal region of the cell. Scale bars, 10 µm. Po: pore plate; S: sulcus; C: cingulum.

*C. monotis* cells were lens-shaped, roundish and compressed anterioposteriorly. The epitheca was slightly smaller than the hypotheca (Figure 3). The nucleus, elongated and slightly curved, was located in the dorsal region of the cell. Cell sizes ranged from 28.14–33.37 µm dorsoventrally and from 27.6–32.62 µm in transdiameter. No differences were observed (*p* > 0.05) between cells in the exponential and stationary growth phases (Table 1). Our findings regarding the *C. monotis* CMBZT14 strain are in agreement with those of Aligizaki and Nikolaidis (2006) [77], Armi et al. (2010) [68], Pagliara and Caroppo (2012) [54] and Ismael (2014) [75].

### 2.3. Molecular Analysis and Phylogeny

**Figure 4:**
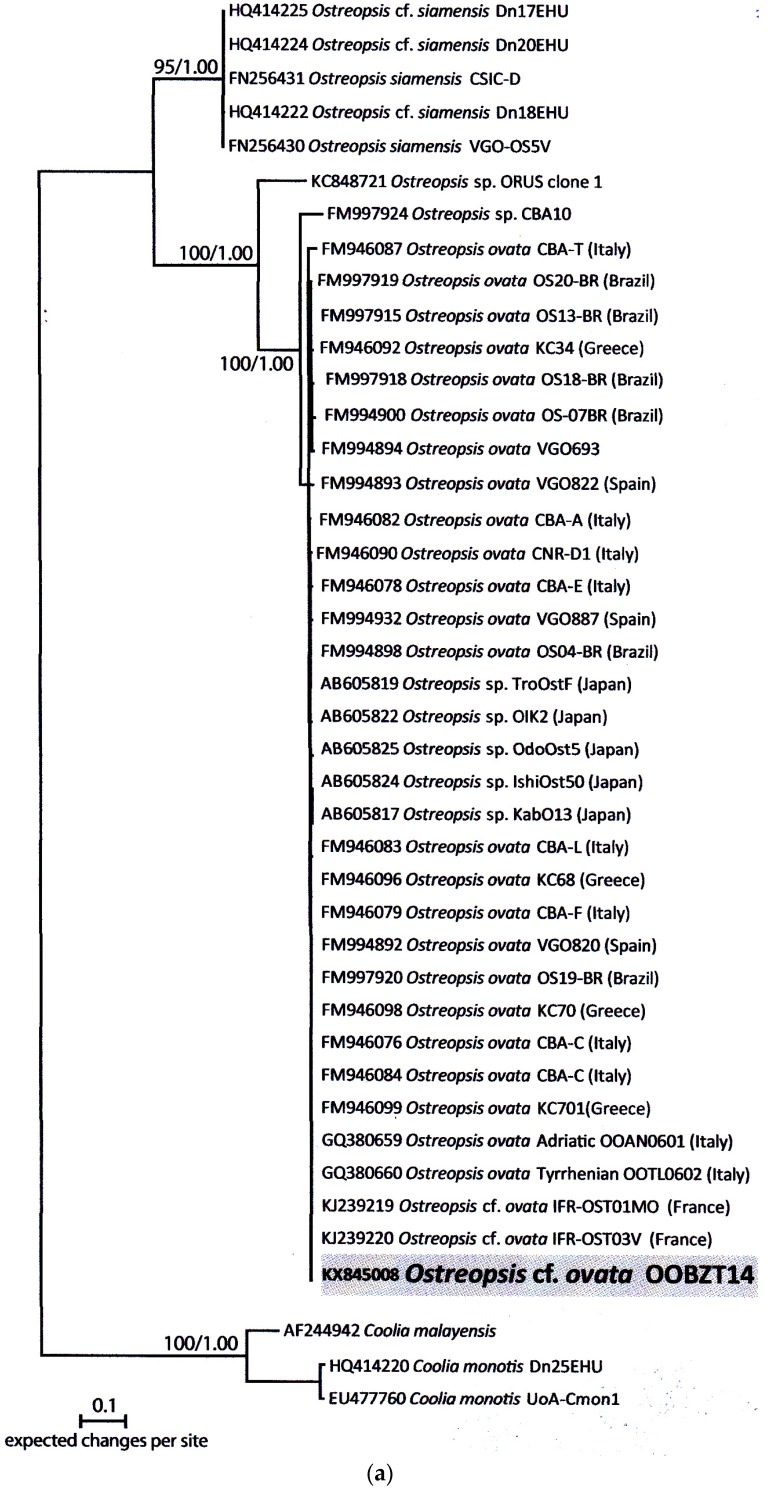

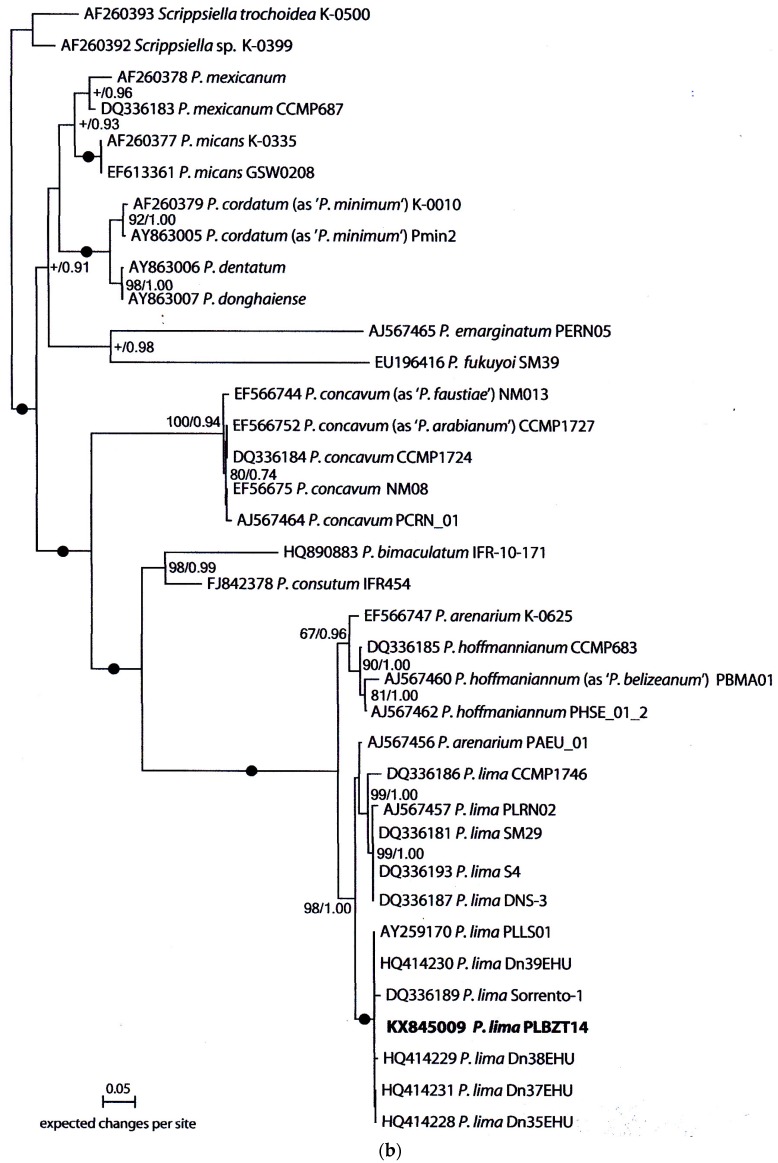

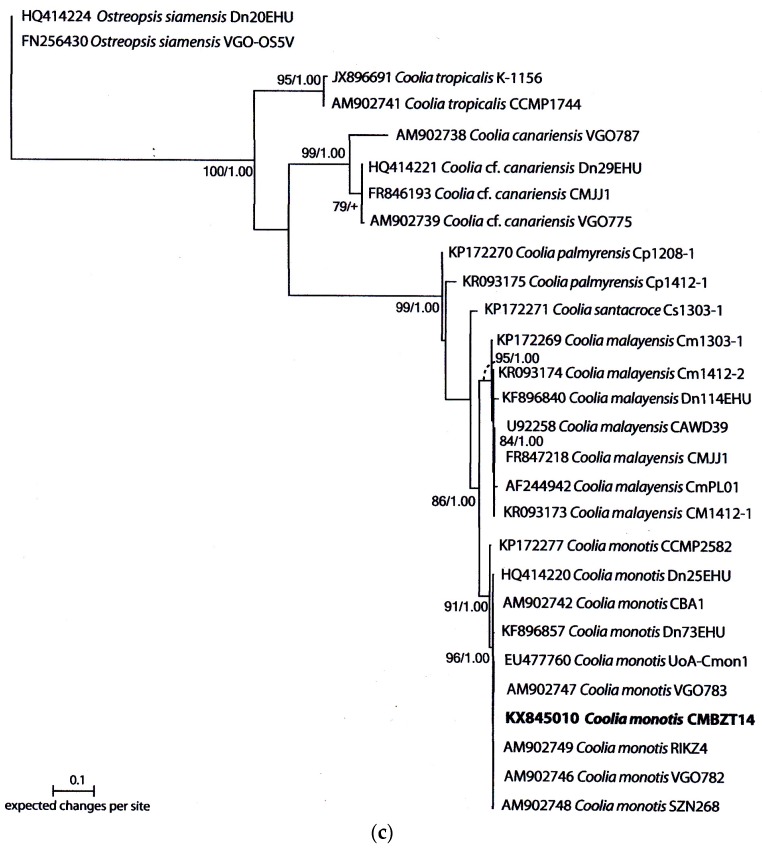
(**a**–**c**) Phylogeny of *Ostreopsis* cf. *ovata* (**a**), *Prorocentrum lima* (**b**) and *Coolia monotis* (**c**) inferred from partial large subunit (LSU) rDNA sequences using maximum likelihood (ML) and Bayesian inference (BI).

Sequences of 930, 856 and 887 base pairs of the partial large subunit (LSU) rDNA (D1-D3) have been obtained from *O*. cf. *ovata* (OOBZT14), *P. lima* (PLBZT14) and *C. monotis* (CMBZT14) strains, respectively. They were deposited in GenBank with Accession Numbers KX845008 (OOBZT14), KX845009 (PLBZT14) and KX845010 (CMBZT14). These sequences were similar to a batch of sequences from France, Italy and Greece for *O.* cf. *ovata*, from Spain, Italy and Australia for *P. lima* and from Greece, Italy and Netherlands for *C. monotis*; all available in GenBank and identified as *O.* cf. *ovata*, *P. lima* and *C. monotis*. The identity of the three strains OOBZT14, PLBZT14 and CMBZT14, determined on the basis of morphological examination of the cells, was then confirmed. The phylogeny inferred from LSU rDNA showed that all of these sequences clustered in a highly supported clade, which indicated that the LSU sequences of the strains from Bizerte Bay are identical to strains mainly found in the Mediterranean Sea and Atlantic Ocean. Results revealed that the OOBZT14 strain was close to strains found in France, Italy and Greece. For *P. lima*, the PLBZT14 strain was identical to strains from Italy and from the Atlantic Spanish coast. The CMBZT14 strain grouped with other stains from Greece and Netherlands. Hence, the three benthic strains from Bizerte Bay belonged to the Mediterranean/Atlantic clades (Figure 4a–c).

### 2.4. Growth Characteristics

#### 2.4.1. *Ostreopsis* cf. *ovata*

The *O.* cf. *ovata* strain reached a maximum cell density of 13,095 cell·mL^−1^ after 24 days of culture. The maximum growth rate was 0.59 ± 0.08 d^−1^. These growth values corresponded to those reported by Granéli et al. (2011) [9] (0.59 ± 0.1 d^−1^), when this species was grown at 28 °C, and by Scalco et al. (2012) [83] (0.55 d^−1^) at 26 °C. Higher growth rates, 0.74–0.83 d^−1^, were also recorded by these authors, but for higher temperature (30 °C) or irradiance (200 µmol photons·m^−2^·s^−1^). The growth rate of the OOBZT14 strain was higher than that found by Guerrini et al. (2010) [22] (0.32–0.37 d^−1^) and Pezzolesi et al. (2012) [88] (0.49 d^−1^) for Mediterranean strains cultivated at 20 °C and much higher than that reported by Nascimento et al. (2012) [89] (0.1–0.15 d^−1^) for Brazilian *O.* cf. *ovata* strains cultivated at a temperature close to that prevailing during our laboratory experiment.

**Figure 5:**
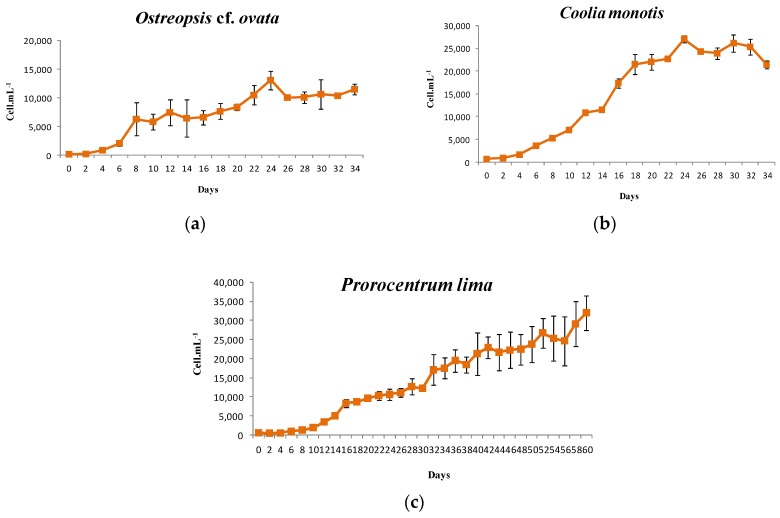
(**a**–**c**) Growth patterns of *Ostreopsis* cf. *ovata* (**a**), *Prorocentrum lima* (**b**) and *Coolia monotis* (**c**) grown in enriched natural sea water medium at a temperature of 25 °C, salinity of 36 and an irradiance of 80 µmol photons·m^−2^·s^−1^ (12L:12D cycle).

In our study, the OOBZT14 growth curve showed a lag phase from Days 0–2, and an exponential phase characterized by three steps: an initial exponential growth from Days 2–8, then a slower growth from Days 8–14, followed by a resumption of growth from Days 14–24. Cells reached the stationary growth phase after 24 days of culture (Figure 5a). Scalco et al. (2012) [83] reported also an exponential growth phase of 20 days, but cultures reaching the stationary phase earlier, after 10 or 13 days, were noted by Guerrini et al. (2010) [22] and Brissard et al. (2014) [19], respectively.

#### 2.4.2. *Prorocentrum lima*

A maximum cell density of 32,019 cell·mL^−1^ was observed after 60 days of culture for *P. lima*. The maximum growth rate was 0.33 ± 0.04 d^−1^. Values, close to ours, were recorded for Pacific strains grown at 28 °C: 0.2–0.35 d^−1^ [34]; and at temperatures ranging from 25 to 29 °C: 0.27 d^−1^ [90]. Lower values were observed for Atlantic strains grown at temperatures ranging from 17–20 °C: 0.092 d^−1^ [91], 0.06–0.14 d^−1^ [39] and 0.11 d^−1^ [40]. In the literature, the highest growth rates for *P. lima* were reported by Morton and Norris (1990) [92] (0.47–0.62 d^−1^) and Tomas and Baden (1993) [93] (0.75 d^−1^) for Atlantic strains growing at high temperatures (27–26 °C) and light intensities (180–150 µmol photons·m^−2^·s^−1^). Thereby, *P. lima* growth seems to be mainly governed by temperature and light conditions.

PLBZT14 was characterized by a quite long lag phase (from Days 0–8) and an exponential growth phase that exceeded 60 days of culture (Figure 5b). A prolonged exponential growth period was noted by Pan et al. (1999) [94] and Varkitzi et al. (2010) [95]. Nevertheless, exponential growth phases not exceeding 16 days [96] or 25 days [40] were also observed.

#### 2.4.3. *Coolia monotis*

For *C. monotis*, the maximum cell density was 27,057 cell·mL^−1^ after 24 days of culture. The maximum growth rate was 0.35 ± 0.01 d^−1^. To our knowledge, studies characterizing the growth potential of *Coolia monotis* are very limited. Faust (1992) [97] reported a doubling time of 3–4 days during the logarithmic phase of growth for cultures grown at 23 °C. Morton et al. (1992) [98] reported growth rates, ranging from 0.2–0.6 d^−1^ for strains grown under different temperatures, salinities and light intensities.

CMBZT14 growth patterns were similar to those of *O.* cf. *ovata*, with a lag phase from Days 0–2 and an exponential phase characterized by three steps: initial exponential growth from Days 2–12, slower growth from Days 12–16 and a resumption of growth from Days 16–24. The beginning of the stationary phase was also observed after 24 days of culture (Figure 5c).

Our results showed that the *O.* cf. *ovata* growth rate (0.59 ± 0.08 d^−1^) is clearly higher than those of *Coolia monotis* (0.35 ± 0.01 d^−1^) and *P. lima* (0.33 ± 0.04 d^−1^), which suggests that *O.* cf. *ovata* has an ecological advantage and can predominate in coastal waters. However, maximum cell densities were inversely proportional to growth rates, with the lowest density recorded for *O.* cf. *ovata* (13,095 cell·mL^−1^) and the highest for *P. lima* (32,019 cell·mL^−1^).

**Figure 6:**
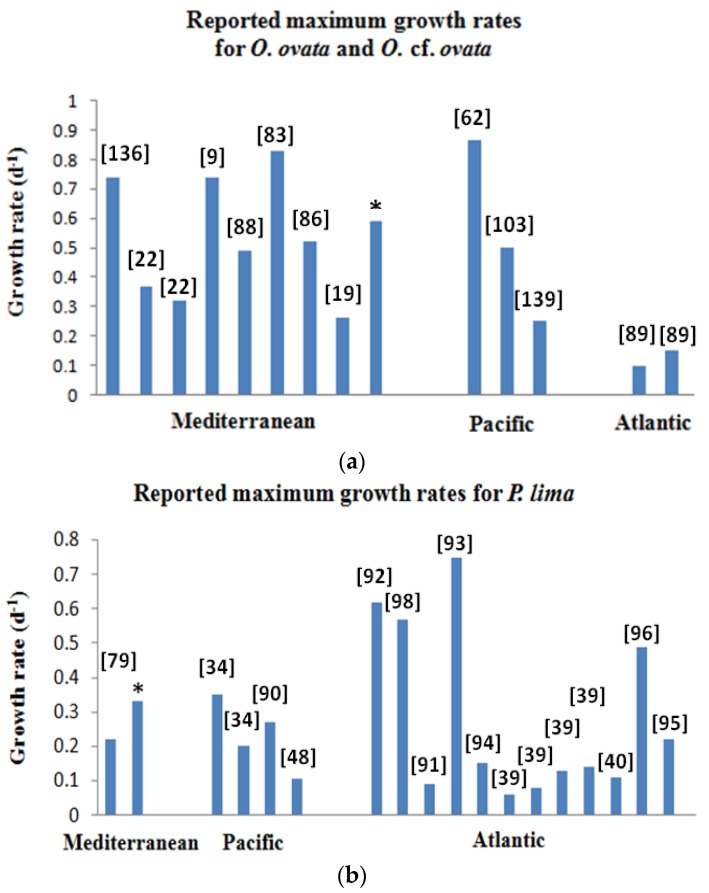
(**a**,**b**) Reported maximum growth rates for *Ostreopsis* cf. *ovata* (**a**) and *Prorocentrum lima* (**b**) in Mediterranean, Pacific and Atlantic waters. References are placed in square brackets. * This study.

Growth characteristics of these dinoflagellates originating from various ecosystems are summarized in Table A1, Table A2 and Table A3. Data are from laboratory experiments of cultured strains growing in different environmental conditions (irradiance, temperature and salinity) corresponding to local conditions. Results from the literature, gathered in these tables and shown in Figure 6, suggest an important variability in the growth of *O.* cf. *ovata* strains colonizing Mediterranean and Pacific waters, with values ranging from 0.25–0.86 d^−1^. Interestingly, *O.* cf. *ovata* strains developing in Atlantic waters showed a lower capacity to grow (0.1–0.15 d^−1^) (Figure 6a). For *P. lima*, the maximum growth rates ranged from 0.06–0.75 d^−1^, with no clear geographic pattern (Figure 6b).

### 2.5. Toxin Profiles

#### 2.5.1. *Ostreopsis* cf. *ovata*

**Figure 7:**
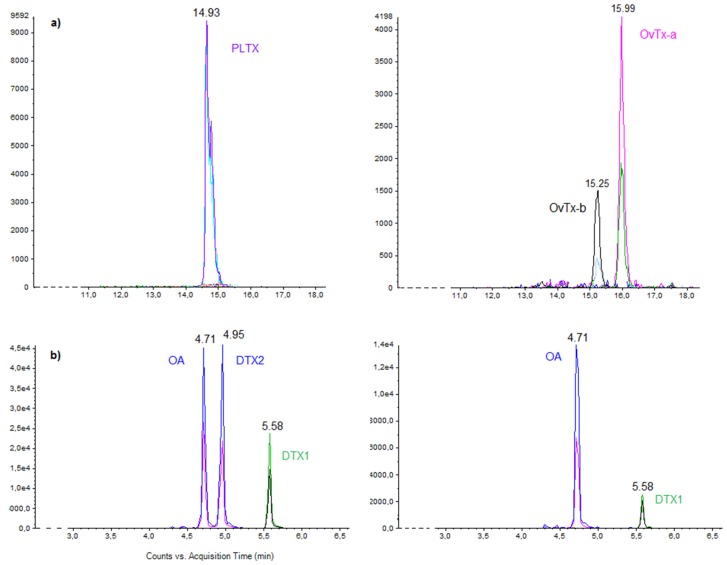

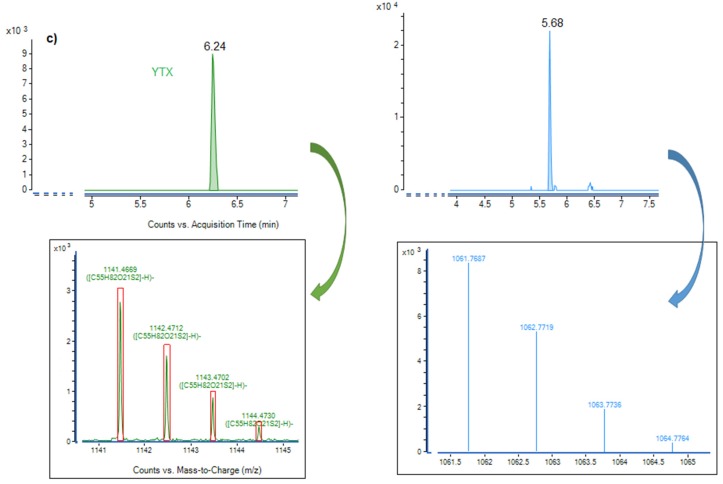
(**a–c**) Liquid chromatography mass spectrometry in tandem (LC-MS/MS) analyses in positive multiple reaction monitoring (MRM) mode for *Ostreopsis* cf. *ovata* (**a**), *Prorocentrum lima* (**b**) and *Coolia monotis* (**c**).

**Figure 8:**
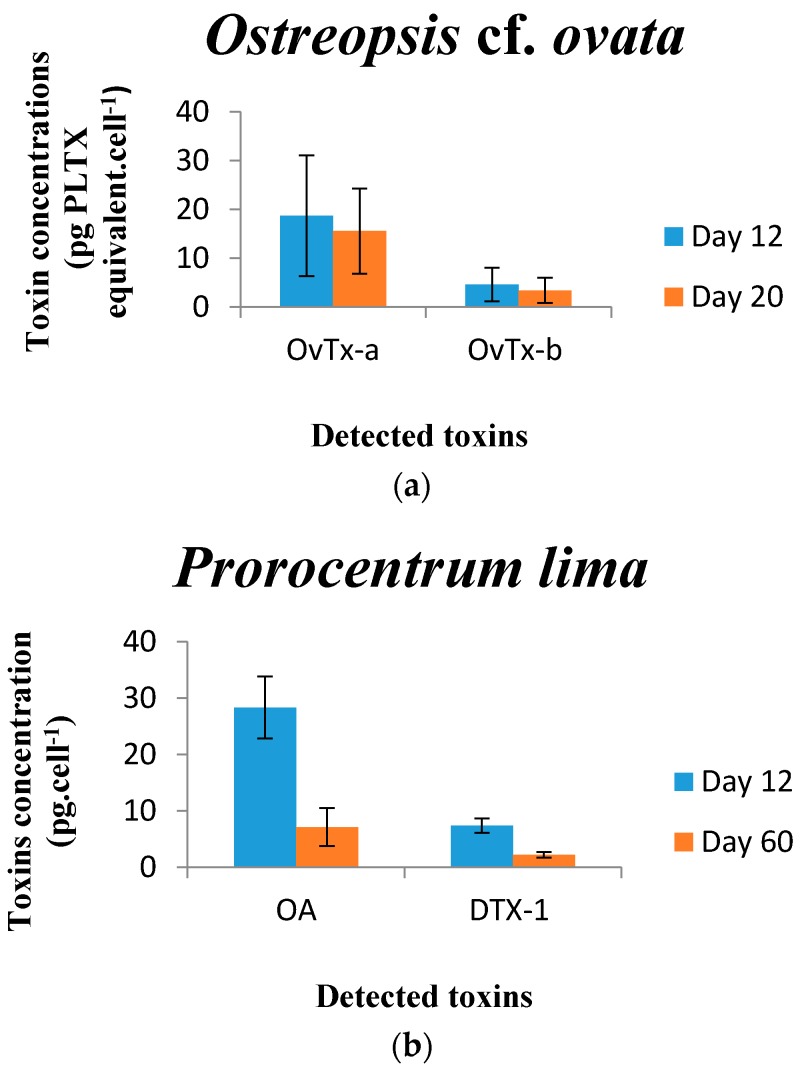
(**a**,**b**) Total amount of toxins measured in cells on Days 12 and 20 for *Ostreopsis* cf. *ovata* and days 12 and 60 for *Prorocentrum lima:* (**a**) ovatoxin-a (OVTX-a) and ovatoxin-b (OVTX-b) in pg PLTX (palytoxin) equivalent·cell^−1^ produced by *Ostreopsis* cf. *ovata*; (**b**) okadaic acid (OA) and dinophysistoxin-1 (DTX-1) in pg·cell^−1^ produced by *Prorocentrum lima*.

Only ovatoxins-a and -b were found in the Tunisian *Ostreopsis* cf. *ovata strain* OOBZT14 (Figure 7a). OVTX-a was the most dominant toxin on Days 12 and 20. A slight decrease in toxin production was observed on Day 20 (OVTX-a = 15.56, OVTX-b = 3.4 pg PLTX equivalent·cell^−1^) in comparison with cells harvested on Day 12 (OVTX-a = 18.7, OVTX-b = 4.6 pg PLTX equivalent·cell^−1^) (Figure 8a). These ovatoxins’ levels are in agreement with those reported for strains from the Adriatic coasts of Italy by Ciminiello et al. (2010) [25] (OVTX-a = 18, OVTX-b = 9 pg·cell^−1^), Vanucci et al. (2012) [85] (OVTX-a = 8.5–19, OVTX-b = 5–11 pg·cell^−1^) and Honsell et al. (2013) [23] (OVTX-a = 7.5–20, OVTX-b = 3.6–9.3 pg·cell^−1^). Lower values were found by Rossi et al. (2010) [28] (OVTX-a = 3.67–9.41, OVTX-b = 1.69–3.43 pg·cell^−1^) and Scalco et al. (2012) [83] (OVTX-a = 2.1–9.81, OVTX-b = 0.7–5.1 pg·cell^−1^) for the Italian strain D483 from the Gulf of Naples. Until now, the highest toxin levels were recorded for the Brazilian isolate LCA-B7 (OVTX-a = 171, OVTX-b = 205 pg·cell^−1^) grown at a temperature of 24 °C and an irradiance of 60 µmol·m^−2^·s^−1^ (12L:12D) [89], for the Spanish strain IRTA-SMM-12-62 (Total toxin content = 250 pg·cell^−1^) grown at a temperature of 24°C and an irradiance of 100 µmol·m^−2^·s^−1^ (12L:12D) [99] and for the French strain IFR-OST-03V (total toxin content = 70–251 pg·cell^−1^) grown at 22 °C and under 420 μmol·m^−2^·s^−1^ (16L:8D cycle) [19].

In our study, no clear pattern was observed for toxin content in relation with growth phase. OOBZT14 cells were harvested at the early (Day 12) and late (Day 20) exponential growth phase. Ovatoxin-a and -b levels decreased slightly after 20 days (Figure 8a). Many reports indicated that *O.* cf. *ovata* increases toxin production during the progression of growth from the exponential to stationary phase [9,22,23,89,100]. Nevertheless, toxin content can vary considerably during each growth phase. Scalco et al. (2012) [83] noted that the cellular toxin content was markedly lower during the post-exponential growth phase than during the exponential phase for cells cultivated at 22 °C and under a 15L:9D illumination cycle. Moreover, based on hemolytic bioassays, Granéli et al. (2011) [9] found that the hemolytic activity on Day 14, was higher than that on Day 20 for *O.* cf. *ovata* cultures growing at 23 °C.

**Figure 9:**
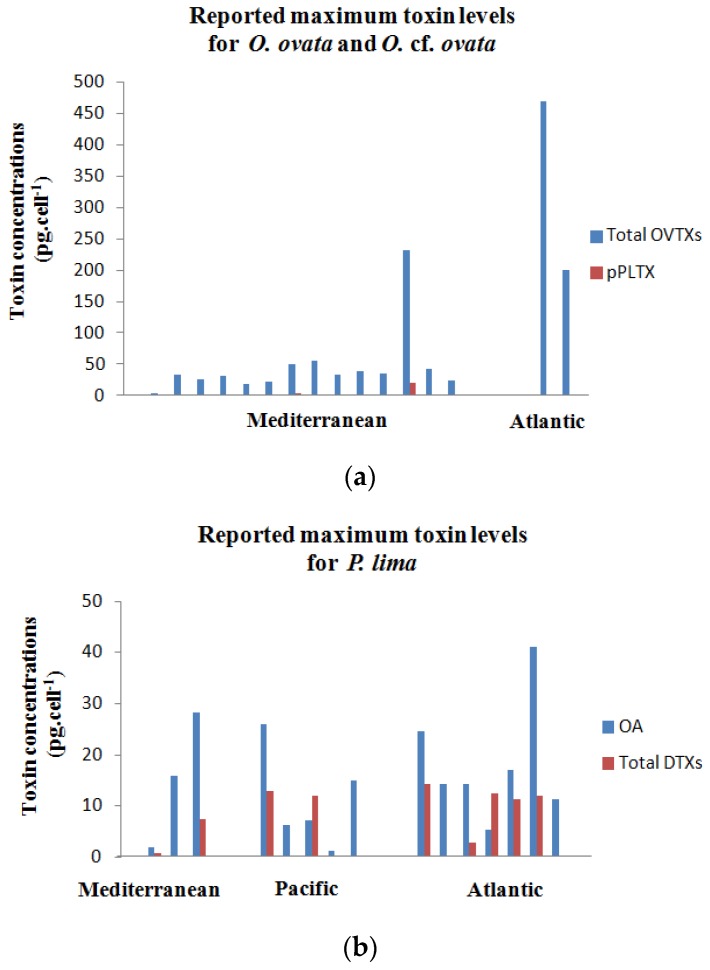
(**a**,**b**) Reported maximum toxin levels for *Ostreopsis* cf. *ovata* (**a**) and *Prorocentrum lima* (**b**) in Mediterranean, Pacific and Atlantic waters.

For *O.* cf. *ovata*, recorded data did not show significant differences in toxin profiles between Mediterranean strains (Table A1). Ovatoxin-a is the predominant toxin except for the Italian strain CBA2-122, which produces higher amounts of ovatoxin-f [27]. In Atlantic waters, Nascimento et al. (2012) [89] found higher levels of OVTX-b than OVTX-a in the Brazilian strains LCA-B7 and LCA-E7. Ovatoxin profiles seem to be strain specific, with isolates that cannot produce some ovatoxins. Our OOBZT14 produced only OVTX-a and -b, and the Italian strain CBA29-2012 was found not to produce OVTX-b and -c [82]. Recently, the presence of a new ovatoxin analog, named ovatoxin-h, was reported for the French strain IFR-OST-03V [65]. Concerning the mascarenotoxins (McTX-a and McTX-c), their presence was only observed for the strain D483 originating from the Gulf of Naples [28,83]. For the putative palytoxin (pPLTX), it is usually found at low levels and is not systematically present in all strains from Mediterranean and Atlantic waters (Figure 9a).

**Figure 10:**
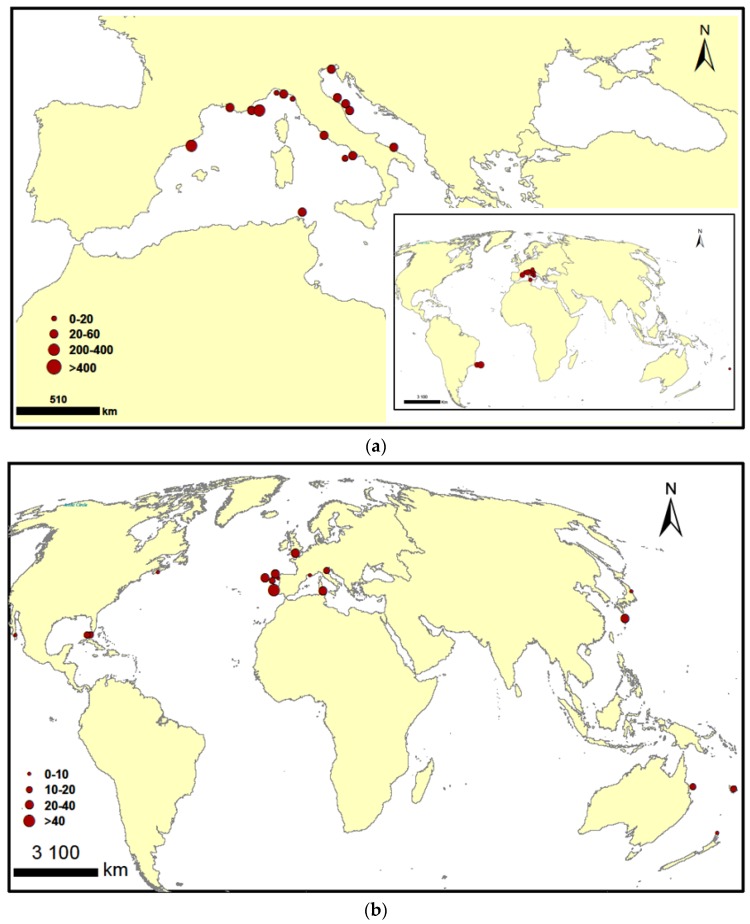
(**a**,**b**) Global distribution of reported cultured toxic strains of *Ostreopsis ovata* (**a**) and *Prorocentrum lima* (**b**). Red dots represent the toxin contents reported for the strains on a per cell basis (pg·cell^−1^); ∑pPLTX + OVTXs for *Ostreopsis ovata* and ∑OA + DTXs for *Prorocentrum lima*.

Concerning toxin production, only two Mediterranean strains (IFR-OST-03V isolated in France and IRTA-SMM-12–62 isolated in Spain) showed high toxic levels (250–300 pg·cell^−1^) [19,99]. All of the other isolates displayed lower toxin contents (3.51–57.5 pg·cell^−1^). Both *O.* cf. *ovata* strains, developing in Atlantic waters, showed high toxin levels (200 and 468 pg·cell^−1^) [89] (Figure 9a and Figure 10a). Few data are available for *Ostreopsis* *ovata* toxicity in Pacific and Indian waters. These strains seem to be non-toxic or to display low toxicity [101,102,103,104]. Rhodes et al. (2010) [105] reported 1.18 palytoxin-equivalents pg·cell^−1^ for an *O. ovata* Pacific strain from Cook Islands (CAWD174). Hemolytic analysis performed by Carnicer et al. (2015) [106] resulted in no palytoxin-like activity in an *O*. cf. *ovata* Indian isolate. Further investigations on Indo-Pacific strains are needed. Nevertheless, *O*. cf. *ovata* toxicity seems to be closely related to their genetics, with toxic strains belonging to the Mediterranean-Atlantic clades and less toxic isolates belonging to the Indo-Pacific clade; even if many other environmental factors are suspected to be involved in toxin production.

#### 2.5.2. *Prorocentrum lima*

Okadaic acid (OA) and dinophysistoxin-1 (DTX-1) were detected in PLBZT14 cells, with the OA being the most predominant toxic compound (Figure 7b). Toxin production was greater for cells harvested on Day 12 (OA = 28.33 pg·cell^−1^, DTX-1 = 7.4 pg·cell^−1^) than after 60 days of culture (OA = 7.13 pg·cell^−1^, DTX-1 = 2.23 pg·cell^−1^) (Figure 8b). PLBZT14 strain seems to be highly toxigenic. Our results are close to those reported by Lee et al. (1989) [31] for Spanish isolates from Vigo (OA = 5–24.5, DTX-1 = 6–14.3 pg·cell^−1^) and a Japanese isolate from Okinawa (OA = 26, DTX-1 = 13 pg·cell^−1^) (Table A2). These OA concentration levels (24.5–26–28.33 pg·cell^−1^) are among the highest found in the literature. Lower maximum values were reported by Nascimento et al., 2005 [40], (OA = 17.13 pg·cell^−1^) for an Atlantic strain and by Holmes et al. (2001) [90] (OA = 15 pg·cell^−1^) for a Pacific isolate. For dinophysistoxin-1, our results are similar to those of Nascimento et al. (2005) [40] (DTX-1 = 0.41–11.29 pg·cell^−1^), Delgado et al. (2005) [107] (DTX-1 = 7.15 pg·cell^−1^) and Bravo et al. (2001) [39] (DTX-1 = 1.01–12.45 pg·cell^−1^). However, trace concentrations and lower amounts of DTX-1 were detected by Tomas and Baden 1993 [93] for an Atlantic strain from Florida and by Barbier et al. (1999) [35] for a Mediterranean isolate from France (DTX-1 = 0.8 pg·cell^−1^).

Concerning the toxin production during growth, maximum toxin concentrations for *P. lima* cells during the stationary phase were described by many authors [39,90,108,109]. At the end of our experiment (Day 60), *P. lima* cells densities are still increasing, and toxin content was lower than after 12 days of culture. Some studies have shown that the toxin production did not increase exponentially during cell growth. Holmes et al. (2001) [90] noted a decrease in OA and 7-deoxy-okadaic acid levels from Days 18–30 and from Days 25–30, respectively, followed by an increase until Day 35. Nascimento et al. (2005) [40] reported that OA and DTX-1 concentrations per cell decreased from Days 1–8, then remained constant during the exponential growth phase, increasing from Days 25–45. In order to draw conclusions, we must determine the toxin production kinetics of OOBZT14 and PLBZT14 throughout the entire growth cycle, by harvesting cells at different growth phases and at different times of a same phase.

For *Prorocentrum lima*, recorded data showed that okadaic acid and dinophysistoxin-1 are the most common compounds in Mediterranean, Atlantic and Pacific strains (Table A2). OA levels are usually higher than those of DTXs (Figure 9b). However, some variability in toxin profiles and production can be observed. Pan et al. (1999) [94] reported the dominance of dinophysistoxin-4 in a Canadian isolate, and Morton and Tindall (1995) [34] found higher methyl-okadaic acid levels in Australian clones from Heron Island. Low DTX-4 and DTX-2 levels were detected for strains from the United Kingdom [40] and from Spain [39], respectively. Prorocentin or 4-hydroxyprorocentrolide and 14-*O*-acetyl-4-hydroxyprorocentrolide were only reported for Pacific strains PL021117001 and PL01 from Taiwan [42,110]. Some OA esters, such as OA-D6, OA-D8 and OA-D9, were found for the strain IO66-01 from Portugal [96]. OA-D10a and OA-D10b were also reported for strains from Southern China [111]. No clear pattern emerges for toxin production as a function of the geographical distribution (Figure 9b). Results from the literature summarized in Table A2 showed for *P. lima* an important variability in the toxin content (0.39–14.3 pg·cell^−1^ for total DTXs and 1.9–41 pg·cell^−1^ for OA), whatever the geographic location considered (Figure 10b).

#### 2.5.3. *Coolia monotis*

For *C. monotis*, Holmes et al. (1995) [52] reported that an Australian isolate contained a monosulfated polyether toxin, named cooliatoxin. A chromatographic peak at 5.6 min was observed in CMBZT14 extracts (Figure 7c). This peak corresponded to a mass close to that of the cooliatoxin. In order to compare the spectra of yessotoxin (YTX) masses and the hypothetical cooliatoxin, an MS/MS fragmentation was performed on samples, as well as on a standard of YTX (Figure 7c). YTX standard was detected at 6.2 min with a mass *m*/*z* = 1141.4669 and an error of 4.2 ppm compared to the calculated mass. The mass spectrum allowed finding the characteristic fragment ions of the molecule at *m*/*z* = 1061.5111, 924.4142, 855.3802 and 713.3157. The detected peak at 5.6 min had a mass *m*/*z* = 1061.768 with an important error of 239 ppm compared to the calculated mass of a mono-sulfated form of YTX. The MS/MS spectrum did not confirm a similar structure to the YTX. In conclusion, for CMBZT14 strain, the major detected peak at 5.6 min with a mass *m*/*z* = 1061.768 close to that of the cooliatoxin (*m*/*z* = 1061.5), did not correspond to a mono-sulfated analogue of the yessotoxin. The structure and toxicity of the unknown compound detected in the CMBZT14 strain needs to be investigated. Fraga et al. (2008) [112] analyzed the toxic content of several *C. monotis* strains (CM2V, CM6V, VGO782, RIKZ4, CCMP1345 and VGO858). A peak at *m*/*z* = 1067 was detected, but the ion was rejected as a YTX analog after complementary mass fragmentation. Observations on other Atlantic and Mediterranean strains confirmed a lack of toxicity in *C. monotis* [20,58,64,113]. Toxic *C. monotis* species were reported by Holmes et al. (1995) [52], Rhodes and Thomas (1997) [56], Rhodes et al. (2000) [55] and Rhodes et al. (2010) [114]. These strains were recently re-identified as *Coolia tropicalis* and *Coolia malayensis* based on the application of molecular techniques [57,115]. Hemolytic activity of *C. monotis* was reported by Nakajima et al. (1981) [53] for Japanese isolates, and Pagliara and Caroppo (2012) [54] showed that the cell lysate of *C.* cfr. *monotis* from Italy had low hemolytic activity and inhibited sea urchin embryo development. However, these studies did not mention genetic data. Further investigations are needed in order to affirm or deny *C. monotis* toxicity. Isolation and characterization of natural compounds from *C. monotis* deserve also more interest, knowing that a ceramide with a novel branched-chain and an unprecedented dioxocyclononane named cooliatin were already identified [116,117].

## 3. Conclusions

*O.* cf. *ovata* (OOBZT14) and *P. lima* (PLBZT14) strains from Tunisian waters are toxic, with ovatoxin-a and okadaic acid being the most abundant compounds, respectively. Tunisian marine ecosystems, as in the whole Mediterranean, are facing water warming. This could promote the development of these thermophilic toxic species. *O.* cf. *ovata*, characterized by a high growth rate in comparison with other benthic species, can out-compete the co-existing microalgae. Blooms of *O.* cf. *ovata* could threaten human health through the emission of noxious aerosols. *P. lima*, characterized by high cell densities and low dispersion capacities, can form toxic hot spots in localized areas and lead to catastrophic effects in the proximity of shellfish farming areas. Measures to protect human health and economic activities must be taken. Monitoring programs have to determine the risk of impacts from toxic benthic microalgae and need to include regular analyses for the related toxins. For *C. monotis* (CMBZT14), further investigations are required to elucidate the chemical structure of the detected compounds and to clarify their toxicological properties by performing mouse bioassays, hemolytic tests and cytotoxicity experiments.

Temperature, salinity and irradiance are the most important environmental factors influencing the growth and cell toxin content of dinoflagellate species [11,118,119,120,121,122,123]. The culture medium and origin of the water used for cultivation could also affect these biological parameters, highlighting specific requirements regarding certain trace elements [124]. The genetic and related physiological plasticity of the strains could also explain the variability of the responses of the dinoflagellates to specified environmental factors [118]. The comparison of the growth and toxin content of *O.* cf. *ovata* and *P. lima* developing in large marine ecosystems, including Mediterranean, Atlantic and Indo-Pacific waters, performed on the basis of data available in the literature, suggests a huge intraspecific variability and that toxin production and growth could be driven by both the intrinsic and the prevailing environmental factors.

## 4. Experimental Section

### 4.1. Sampling Site

**Figure 11:**
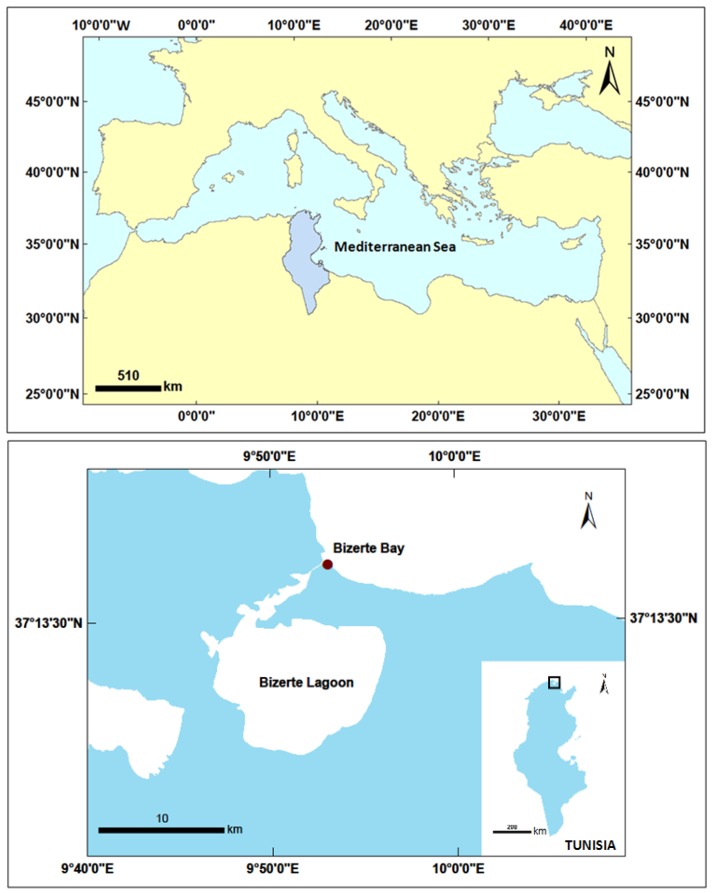
Map of the location of the Bizerte Bay (North of Tunisia, Southern Mediterranean).

*Ostreopsis* cf. *ovata*, *Prorocentrum lima* and *Coolia monotis* were collected from Bizerte Bay, North of Tunisia (37°16′7 N 9°52′58 E), in April and July 2014 (Figure 11). Bizerte Bay is situated in a harbor area and is connected to a semi-enclosed lagoon, the Bizerte Lagoon. Several oyster and mussel farms are implemented in this coastal lagoon, which represents one of the major aquaculture areas in Tunisia. Sporadic HABs events were recorded in Bizerte lagoon in association with PSP (*Alexandrium* spp.), DSP (*Dinophysis* sp., *P. lima*, *P. mexicanum* and *P. minimum*) and ASP (*Pseudo-nitzschia* spp.) episodes [125]. *P. lima* represents a significant part of the seawater microphytoplanktonic community of the Bizerte Lagoon, reaching concentrations higher than 10^4^ cell·L^−1^ [69]. No published data are available for the Bizerte Bay. Nonetheless, this bay can shelter toxic dinoflagellates, which can increase their range via the channel and proliferate in the lagoon.

### 4.2. Isolation and Culture Conditions

The three benthic dinoflagellates were isolated from the macrophyte *Cymodocea nodosa.* Fresh leaves of this magnoliophyte were hand-collected in Bizerte Bay (0.5–1-m depths), placed in plastic jars containing seawater and transported to the laboratory. Twenty grams of leaves were placed into a jar containing 250 mL of seawater (previously filtered through 180 μm) and vigorously shaken to allow the dislodgement of epiphytic microalgal cells. The sample was then concentrated on a 20-µm mesh sieve and observed under an inverted photonic microscope. Cells were harvested in April 2014 for *P. lima* and *C. monotis* and July 2014 for *O.* cf. *ovata*. The three strains were isolated by picking a large number of single cells using the capillary pipette method. Non-axenic monoclonal cultures were grown in enriched natural sea water medium (ENSW) [126], at stable conditions of salinity 36, temperature 25 °C and irradiance 80 µmol photons·m^−2^·s^−1^ in a 12:12 light:dark cycle. The strains were named OOBZT14, PLBZT14 and CMBZT14 corresponding to *O.* cf. *ovata*, *P. lima* and *C. monotis*, respectively.

### 4.3. Morphology

Morphometric features were determined using a photonic microscope (Leica microsystems CMS GmbH, DM IL LED model, Wetzlar, Germany). Vegetative cells in exponential and stationary growth phases were fixed and cell dimensions determined at 400× magnification using Leica Application Suite software (LAS, Version 3.0, Leica Microsystems Ltd, Heerbrugg, Switzerland). For each strain, the length and width of up to 30 cells were measured. To determine the thecal plate morphology, cells were stained with calcofluor (0.5 mg·mL^−1^, Sigma-Aldrich, St. Louis, MO, USA) [127] and observed under a Leica epifluorescent microscope (Leica microsystems CMS GmbH, DM2500 M model, Wetzlar, Germany). DAPI staining was also performed to observe nuclear DNA. The identification of the three benthic dinoflagellates was then confirmed by ribotyping.

### 4.4. Molecular Analysis and Phylogeny

#### 4.4.1. DNA Extraction and PCR

Total genomic DNA was extracted from the pellets of the three strains (OOBZT14, PLBZT14 and CMBZT14) obtained by centrifuging cultures of 30 mL during 10 min at 3500× *g* and 4 °C. For the extraction, the classical phenol-chloroform method was used [128]. The cellular material was released by enzymatic lysis, using proteinase K digestion. The DNA was separated from protein by phenol:chloroforme:isoamyl alcohol (25:24:1) extraction, then extracted using chloroform:isoamyl alcohol (24:1). The separation of the aqueous and organic phases was performed by centrifugation. The aqueous phase contains the DNA, which was ultimately recovered in solid form, as a result of precipitation in ethyl alcohol. DNA was then resuspended on ultra-pure water. For PCR, the oligonucleotide primers and methods used were those described in Nézan et al. (2014) [129]. We focused on Internal Transcribed Spacers ITS regions and D1–D3 areas of the 28S rRNA of the strains, since these regions have been shown to be efficient to discriminate species.

All reagents were purchased from Sigma-Aldrich, St. Louis, MO, USA.

#### 4.4.2. Phylogeny

For the phylogenetic analyses, the sequences of the Bizerte bay strains were aligned together with other related sequences in three independent datasets. For *Ostreopsis* cf. *ovata*, a matrix of 684 bp and 42 LSU rDNA sequences including the strain OOBZT14 and 38 other *Ostreopsis* sequences and 3 sequences of *Coolia* (as the outgroup) retrieved from GenBank was used. For *Prorocentrum lima*, a matrix of 977 bp and 36 sequences including PLBZT14 strain and 33 sequences of *Prorocentrum* and two sequences of *Scrippsiella* (as the outgroup) retrieved from GenBank was used. For *Coolia monotis*, a matrix of 581 bp and 28 LSU rDNA sequences including the strain CMBZT14 and 25 other *Coolia* sequences, and two sequences of *Ostreopsis* (as the outgroup) retrieved from GenBank was prepared. The matrixes of *Ostreopsis* and *Coolia* sequences were aligned using MAFFT software Version 7 [130], with selection of the Q-ins-i algorithm, which considers the secondary structure for the alignment, while the *Prorocentrum* sequences were aligned using MUSCLE software v. 3.7 [131]. The three alignments were refined by eye and analyzed by two methods of phylogenetic reconstruction: maximum likelihood (ML), using PhyML v.3.0 software [132] and Bayesian inference (BI) using MrBayes v. 3.1.2 [133]. The software jModeltest v 0.1.1 [134] was first used to select the most suitable model of substitutions. The general-time reversible model (GTR + I + G) was chosen as indicated by the hierarchical likelihood ratio tests (hLRTs), Akaike Information Criterion 1 (AIC1), Akaike Information Criterion 2 (AIC2) and Bayesian information criterion (BIC) tests implemented in jModeltest. Bootstrap values (support for branches) of trees were obtained after 1000 iterations in ML. For Bayesian inference, four Markov chains were run simultaneously for 2 × 10^6^ generations with sampling every 100 generations. On the 2 × 10^4^ trees obtained, the first 2000 were discarded (burn-in), and a consensus tree was built from the remaining trees. The posterior probabilities corresponding to the frequency, with which a node is present in preserved trees, were calculated using a coupled Monte Carlo Metropolis approach-Markov Chain (MCMC).

### 4.5. Growth Characteristics

For each strain, cell concentrations were followed in triplicates, every two days, during 34 days for *O.* cf. *ovata* and *C. monotis* and during 60 days for *P. lima*. After homogenization, 3 mL of each culture were taken out axenically from the 250-mL flasks, always at the same time of the day. Fixed cells were counted on a Sedgewick-Rafter counting slide, under an inverted photonic microscope. In accordance with Guillard [135], the maximum growth rate (μ_m_; expressed in day^−1^) was calculated from the slope of a linear regression over the entire exponential phase of growth by the least square fit of a straight line to the data after logarithmic transformation; μ_m_ = Ln (*N*_1_) − Ln (*N*_0_)/*T*_1_ − *T*_0_ in units of day^−1^, where *N*_1_ and *N*_0_ were the cell density at time *T*_1_ and *T*_0_, respectively, during the linear portion of the exponential growth phase.

### 4.6. Toxin Analysis

Twenty eight milliliters of the corresponding culture were harvested at Days 12 and 20 for *O*. cf. *ovata* and *C. monotis* and at Days 12 and 60 for *P. lima*. Cells were centrifuged at 3500× *g* during 10 min and the supernatant carefully removed. The pellets were stored at −20 °C until toxin analysis.

#### 4.6.1. Sample Preparation

Culture pellets were dissolved with 1 mL methanol 100% for *P. lima* and *C. monotis* and with 1 mL methanol 90% for *Ostreopsis* cf. *ovata.* Mixtures were ground with glass beads (0.25 g) in a mixer mill (Retsch MM400, Germany) for 30 min. After centrifugation at 5000× *g* during 10 min, the supernatants were collected and filtered through 0.2 µm before injection to the liquid chromatograph and mass spectrometry in tandem (LC-MS/MS).

#### 4.6.2. Instrumentation: LC-MS/MS Systems

The analyses were carried out using two LC-MS/MS systems: (A) triple quadripole (QqQ); (B) high resolution quadrupole time of flight (Q-TOF). For the liquid chromatography conditions, a C_18_ Kinetex column (Phenomenex, Torrance, CA, USA) was employed with a linear gradient using water as Eluent A and 95% acetonitrile/water as Eluent B, both eluents containing 2 mM ammonium formate and 50 mM formic acid.

System A is composed of an LC system (UFLC Nexera, SHIMADZU, Tokyo, Japan) coupled to a hybrid triple quadrupole/ion-trap mass spectrometer (API4000Qtrap, SCIEX, Redwood City, CA, USA) equipped with a Turbospray^®^ interface (SCIEX, Redwood City, CA, USA). The instrument control, data processing and analysis were conducted using Analyst software. Mass spectrometry detection was performed in both negative and positive mode using multiple reaction monitoring (MRM) and scanning a minimum of two transitions for each toxin.

System B is composed by a UHPLC system (1290 Infinity II, Agilent Technologies, Santa Clara, CA, USA) coupled to a 6550 ifunnel Q-TOF (Agilent Technologies, Santa Clara, CA, USA) equipped with a Dual Jet Stream™ (Agilent Technologies, Santa Clara, CA, USA) -ESI source. The instrument was operated in full scan and targeted MS/MS mode. The experiments were acquired in negative or positive, depending on the compound ionization.

The mass spectra were acquired over the *m*/*z* 100–1700 range with an acquisition rate of 2 spectra/s. The targeted MS/MS mode was applied over the *m*/*z* 50–1700 range with an MS scan rate at 10 spectra/s and an MS/MS scan rate at 3 spectra/s. Three fixed collision energies (20, 40 and 60 eV) were applied to the precursor ions to obtain an overview of the fragmentation pathways. The instrument control, data processing and analysis were conducted using Mass Hunter software (Agilent technologies, Santa Clara, CA, USA).

## Figures and Tables

**Table 1:** Morphometric characteristics of *Ostreopsis* cf. *ovata* (OOBZT14), *Prorocentrum lima* (PMBZT14) and *Coolia monotis* (CMBZT14) strains: mean, minimum, maximum values (µm) and standard deviation of the length and width of the cells harvested in both exponential and stationary growth phases (*n* = 30).

Growth Phase Measures		Exponential Phase		Stationary Phase	
		Length (µm)	Width (µm)	Length (µm)	Width (µm)
***O.* cf. *ovata***	Mean	**50.38**	**36.80**	**55.18**	**39.58**
	SD	4.36	3.33	5.25	3.61
	Min	41.85	32.92	42.90	36.12
	Max	58.51	45.28	65.01	45.88
***P. lima***	Mean	**45.69**	**36.00**	**45.45**	**36.04**
	SD	1.66	1.35	1.88	1.66
	Min	42.98	34.94	42.02	33.61
	Max	48.80	37.95	49.13	37.34
***C. monotis***	Mean	**30.65**	**29.23**	**30.66**	**29.35**
	SD	1.32	1.44	2.77	3.27
	Min	28.14	27.63	23.13	22.07
	Max	33.37	32.62	35.58	36.96

## Abbreviation

The following abbreviations are used in this manuscript:

Opt	Optimum
Dt	Doubling time
µmax	Maximum growth rate
S-PES	Seawater with Provasoli’s ES supplement
S-ES-1	Seawater with ES-1supplements

## Appendix A

**Table A1:** Summary for *Ostreopsis ovata* established laboratory cultures from various marine ecosystems. Culture conditions, growth rates and toxicity are specified when available.

Strain and Sampling Location	Temperature (°C)	Salinity	Irradiance (µmol photons.m^−2^·s^−1^) and L:D Cycle (h)	Culture Medium	Growth Rate (d^−1^)	Toxicity: Detected Toxins (pg·cell^−1^), Hemolytic Activity, Toxicity to Mice and Other Organisms	Isolated from	Reference
**MEDITERRANEAN WATERS**								
CNR-A1 (Italy, Tyrrhenian Sea, Gioia Tauro)	17 ± 1	**	100 (14L:10D)	K, F/20, F/2	**	Presence of palytoxin Hemolytic activity on human erythrocytes	Seawater *Rhodophyceae Phaeophyceae*	Penna et al. (2005) **^b^** [20]
CNR-D1 (Italy, Tyrrhenian Sea, La Spezia)								
CNR-Z1 (Spain, Balearic Sea, Paguera)								
** (Greece, North Aegean coasts)	19 ± 1	**	70 (14L:10D)	F/2, K	**	**	Macrophytes	Aligizaki and Nikolaidis (2006) **^b^** [77]
** (Italy, Ligurian coast, Genoa)	25	**	2000 lX (16L:8D)	K-Keller	**	OVTX-a = 3.11/3.85, pPLTX = 0.40/0.55	*Rhodophyta, Chlorophyta, Phaeophyta*	Ciminiello et al. (2008) **^b^** [24]
D483 (Italy, Gulf of Naples, Gaiola)	18	**	50 (12L:12D)	K/2	**	OVTX-a = 3.67–9.41, OVTX-b = 1.69–3.43, OVTX-c = 2.51–4.12, OVTX-d = 0.08–0.74, Mascarenotoxin-a = ND-0.47, Mascarenotoxin-c = ND-0.32	*Asparagopsis taxiformis*	Rossi et al. (2010) **^a^** [28]
VGO820, VGO1049 (Spain, Catalonian coast, Llavaneres)	20	**	174.4 (10L:14D)	K/2, K, L1, Schreiber	0.49–0.74	**	Seawater	Bravo et al. (2010) **^b^** [136]
OOAN0601 (Italy, Adriatic coast, Marche region, Numana)	20	32	90 (16L:8D)	F/2	**	OVTX-a = 18, OVTX-b = 9, OVTX-c = 2, OVTX-d+e = 4, pPLTX = 0.2	Seawater (proximity of *Cystoseira sp.* and *Alsidium corallinum*)	Ciminiello et al. (2010) **^b^** [25]
OOTL0602 (Italy, Tyrrhenian Sea, Lazio region, Porto Romano)	20	35	90 (16L:8D)	F/2	0.32	≈OVTX-a = 14–25, pPLTX = 0.7–1.1 (in cells)	Seawater (proximity of *Cystoseira sp*. and *Alsidium corallinum*)	Guerrini et al. (2010) **^b^** [22]
OOAN0601 (Italy, Adriatic Sea, Marche region, Numana)	20	35	90 (16L:8D)	F/2	0.37	≈OVTX-a = 18.5–31, pPLTX = 1.3–2.5 (in cells)	Seawater (proximity of *Cystoseira sp*. and *Alsidium corallinum*)	Guerrini et al. (2010) **^b^** [22]
KAC85 (Italy, Tyrrhenian Sea, Monte Argentario)	16–24, 26,28,30	38	140 (16L:8D)	F/10	0.1–0.74	Hemolytic activity on horse blood cells 20 °C = 18.1 ng·SnE·cell^−1^. 22 °C = 11.57 ng·SnE·cell^−1^. (saponin nano-equivalent per cell)	Seaweeds	Granéli et al. (2011) **^b^** [9]
** (Italy, Northern Ionian Sea, Mar Piccolo, Mar Grande and Lido Bruno)	24 ± 2	37	100 (12L:12D)	F/2	**	Live cells: strongly affected *P. lividus* embryonic development.Cell lysate: inhibited *P. lividus* embryonic development, were toxic to *Artemia salina* nauplii and induced hemolysis on human erythrocytes	Seawater Rocks scraping	Pagliara and Caroppo (2012) **^c^** [54]
OOAN0601 (Italy, Adriatic coast, Marche region, Numana)	20,25,30	26,32,36,40	90,100–110 (16L:8D)	F/2	0.34–0.49	OVTX-a, -b, -c, -d, -e, pPLTX. Total toxin content in cell pellets = 57–155 (µg·L^−1^) Hemolytic activity on sheep erythrocytes Toxic to *Artemia sp*. nauplii and to *D. labrax*	Seawater (proximity of *Cystoseira sp.* and *Alcidium corallinum*)	Pezzolesi et al. (2012) **^a^** [88]
D483 (Italy, Gulf of Naples) CBA-T (Italy, Portonovo) OS2T (Italy, Gulf of Trieste)	18,22,26,30	36	50–200 (9L:15D, 12L:12D, 15L:9D)	K/2	0.18–0.83	Strain D483: OVTX-a = 2.1–9.81, OVTX-b = 0.7–5.1, OVTX-c = 0.005–1.2, OVTX-d+e = 0.22–6.8, McTX-a = 0.006–0.47, McTX-c = ND-0.32	**	Scalco et al. (2012) **^a^** [83]
IFR-OST-0.1M (France, Marseille, Frioul Island, Morgiret)	22	35	460 (16L:8D)	K, L1	**	OVTX-a = 50, pPLTX = 3.7	Seawater (proximity of *Dictyota sp.* and *Haliptilon virgatum*)	Sechet et al. (2012) **^a^** [61]
IFR-OST-0.1V (France, Villefranche-sur-Mer Bay)	22	35	460 (16L:8D)	K, L1	**	OVTX-a = 55, pPLTX = 2.5	Seawater (dominant algae *Halopteris scoparium)*	Sechet et al. (2012) **^a^** [61]
CBA2-122 (Italy, Adriatic Sea, Portonovo)	23 ± 1	**	100 (14L:10D)	F/4	**	OVTX-f = 17, OVTX-a = 8, OVTX-b = 6, OVTX-c = 0.8, OVTX-d+e = 2, pPLTX = 0.1	**	Ciminiello et al. (2012) **^a^** [27]
OOAN0918 (Italy, Adriatic Sea, Passetto)	20	36	90 (16L:8D)	F/2	**	**≈** OVTX-a = 8.5–19, OVTX-b = 5–11, OVTX-c = 1–2, OVTX-d+e = 3–6, pPLTX = 0.5–1	Seawater	Vanucci et al. (2012) **^a^** [85]
C5 (Italy, Adriatic Sea, Gulf of Trieste, Canovella de’ Zoppoli)	23 ± 1	**	100 (14L:10D)	F/4	**	OVTX-a = 7.5–20, OVTX-b = 3.6–9.3, OVTX-c = 0.6–1.5, OVTX-d+e = 1.6–4.4, pPLTX = 0.03–0.08	Seawater	Honsell et al. (2013) **^a^** [23]
OOAB0801 (Italy, Adriatic Sea, Puglia region)	20 ± 1	36	110–120 (16L:8D)	F/2	0.52	OVTX-a = 52%–55%, OVTX-b = 25%–29%, OVTX-c = 4%–7%, OVTX-d+e = 11%–16%, pPLTX = 1%–2%. Maximum PLTXs content = 21.5	**	Pezzolesi et al. (2014) **^a^** [86]
IFR-OST-03V (France, Villefranche sur-Mer)	22	35	420 (16L:8D)	L1	0.26	OVTX-a = 50%, OVTX-b = 25%, OVTX c = 9%, pPLTX = 8%, OVTX-d = 4%, OVTX-e = 3%, OVTX-f = 1%. Toxin content = 70–251	Seawater (proximity of *Stypocaulon sp*. and *Acetabularia sp*.)	Brissard et al. (2014) **^a^** [19]
IFR-OST-03V (France, Villefranche-sur-Mer)	22	38	420 (16L:8D)	L1	**	Ovatoxin-h, OVTX-a,-b, -c, -d, -e,-f, pPLTX	Seawater (proximity of *Stypocaulon sp*. and *Acetabularia sp*.)	Brissard et al. (2015) **^a^** [65]
6 strains including IRTA-SMM-12-62 (Spain, South Catalonia, Ebro River Delta)	24	36	100 (12L:12D)	**	**	OVTX-a,-b, -c, -d, -e,-g, IsobPLTX Total toxin content = 50–250	*Jania rubens*	García-Altares et al. (2015) **^a^** [99]
CBA29-2012 (Italy, Quarto dei Mille Genoa)	20 ± 0.5	**	85–135 (16L:8D)	F/2	**	OVTX-a = 33.5, OVTX-d+e = 9, pPLTX = 1.5. Total toxin content in cell pellets = 44Toxic to *Artemia salina* nauplii	****	Giussani et al. (2015) **^a^** [82]
OOBZT14 (Tunisia, Bizerte Bay)	25	36	80 (12L:12D)	ENSW	0.59	OVTX-a = 15.56–18.7, OVTX-b = 3.4–4.6	*Cymodocea nodosa*	This study **^a^**
**ATLANTIC WATERS**								
Isolate 538 (Carribean Sea, Leeward Islands, IIe St. Barthelemy, Port de Gustavia)	28	35	300 ft-c (12L:12D)	GPM	**	Mouse bioassay: lack of water or lipid soluble ciguatera toxins in *O. ovata* extracts	Tide-pool	Besada et al. (1982) **^b^** [137]
IEO-OS06BR, IEO-OS15BR (Brazil, Rio de Janeiro)	17 ± 1	**	100 (14L:10D)	K, F/20, F/2	**	Presence of palytoxin Hemolytic activity on human erythrocytes	*Rhodophyceae Phaeophyceae*	Penna et al. (2005) **^b^** [20]
LCA-B7 (Brazil, Rio de Janeiro, Armação dos Búzios)	24 ± 2	**	60 (12L:12D)	L2/2	0.1	OVTX-a = 78–171, OVTX-b = 87–205, OVTX-c = 3–37, OVTX-d+e = 5–55 Hemolytic activity on rabbit erythrocytes	*Sargassum vulgare* bed	Nascimento et al. (2012) **^a^** [89]
LCA-E7 (Brazil, Rio de Janeiro, Armação dos Búzios)	24 ± 2	**	60 (12L:12D)	L2/2	0.15	OvTx-a = 20–71, OvTx-b = 23–77, OvTx-c = 4–30, OvTx-d+e = 3–80 and pPLTX = ND-0.62 Hemolytic activity on rabbit erythrocytes	*Sargassum vulgare* bed	Nascimento et al. (2012) **^a^** [89]
Dn145EHU, Dn146EHU, Dn147EHU (Portugal, Lagos)	20	**	80 (12L:12D)	F/4	**	**	Seawater Macroalgae	David et al. (2013) **^a^** [138]
**PACIFIC WATERS**								
** (Japan, Okinawa, Ishigaki Island)	25	**	4000–8000 lx (18L:6D)	S-PES	**	Butanol soluble fraction toxic to mice Hemolytic activity on mouse blood cells No effects on killifish	*Turbinaria ornata Amphiroa sp*.	Nakajima et al. (1981) **^b^** [53]
CAWD174 (Cook Islands, south coast of Rarotonga)	**	**	**	F/2	**	Palytoxin-equivalents = 1.18 Not Toxic to miceNegatif haemolysis neutralisation assay	*Halimeda sp.*	Rhodes et al. (2010) **^b^** [105]
s0715, s0662 (Japan, Kochi, Subogata and Tei)	25	**	100 (12L:12D)	PES, F/2, IMK	**	Toxic to mice	*Tricleocarpa Pterocladiella, Dictyota*	Sato et al. (2011) **^a^** [102]
s0662 (Japan, Kochi, Tei)	25	30.8	90–100 (12L:12D)	F/2, IMK, PES, SWM3	0.181–0.866	**	**	Yamaguchi et al. (2012) **^a^** [62]
s0662 (Japan, Kochi, Tei)	24–30	31	140 (12L:12D)	F/10	≈0.22–0.5	Haemolytic activity on horse blood cells only during the decaying phases 25 °C = 0.70 ± 0.15 SnE cell−1 27 °C = 0.46 ± 0.01 SnE cell−1 (ng saponin equivalent per cell)	**	Vidyarathna and Granéli (2012) **^b^** [103]
JHAOS5, JHWOS13 (Korea, Jeju Island)	20	30	180 (12L:12D)	IMK, F/2	0.15–0.25	Strain JHAOS5: Supressed the growth of HL-60 cells (=human promyelocytic leukemia tumor cell line)	Sand, Macroalgae	Shah et al. (2014) **^b^** [139]
**INDIAN WATERS**								
P-0117, P-0128 (Reunion Island, East coast, West Indian Ocean)	26	**	20–40 (12L:12D)	F/2	**	Haemolytic analysis (sheep blood):no palytoxin-like activity	*Actinotrichia fragilis, Turbinaria conoides, Jania* sp., *Galaxaura* sp	Carnicer et al. (2015) **^a^** [106]
**INDO-PACIFIC WATERS**								
20 strains (Peninsula and East of Malaysia)	26–27	32	30 (12L:12D)	ES-DK	**	4 strains/20 toxic to Artemia franciscana, 16 strains not toxic	*Sargassum spp. Padina spp*. Seagrasses Dead corals	Mohammad Noor et al. (2007) **^b^** [101]
TD7OS, TF5OS (Gulf of Thailand and Andaman Sea)	25	31 ± 1	** (12L:12D)	IMK/2	**	Both strains did not cause the death of mice, abnormal behavior observed.	*Padina spp. Sargassum spp*	Tawong et al. (2014) **^a^** [104]

****** No Data; ≈ Seen in figures; *^a^ O.* cf. *ovata*; *^b^ O. ovata*; *^c^ O.* cfr. *ovata*. Cellular toxin content is expressed in pg·cell^−1^ or other specified unit. Irradiance is expressed in µmol photons·m^−2^·s^−1^ or other specified unit; Abbreviations: S-PES = Seawater with Provasoli’s ES supplement; PES = Provasoli enriched seawater, ND = Not detected.

**Table A2:** Summary for *Prorocentrum lima* established laboratory cultures from various marine ecosystems. Culture conditions, growth rates and toxicity are specified when available.

Strain and Sampling Location	Temperature (°C)	Salinity	Irradiance (µmol photons.m^−2^·s^−1^) and L:D Cycle (h)	Culture Medium	Growth Rate (d^−1^)	Toxicity: Detected Toxins (pg·cell^−1^), Hemolytic Activity, Toxicity to Mice and Other Organisms	Isolated from	Reference
**MEDITERRANEAN WATERS**								
MARS1 (France, Marseille)	20	**	40 (12L:12D)	F/2	**	OA = 1.9, DTX-1 = 0.8	**	Barbier et al. (1999) [35]
Several strains including KC2, KC6, KC45, KC49, KC60 (Greece, North Aegean coasts)	19 ± 1	**	70 (14L:10D)	F/2	**	PP2AIA (Protein phosphatase type 2A inhibition assay):Estimated OA equivalents > 0.50–10.23 KC2, KC6: Toxic to *Artemia* nauplii	Macrophytes	Aligizaki et al. (2009) [87]
** (Italy, Adriatic Sea, lagoon of Goro)	20	25	90 (16L:8D)	F/2	0.22,0.23	OA = 6.69–12.50/15.8, DTX-1 = 0.12–0.39	**	Vanucci et al. (2010) [79]
PLBZT14 (Tunisia, Bizerte Bay)	25	36	80 (12L:12D)	ENSW	0.33	OA = 7.13–28.33, DTX-1 = 2.23–7.4	*Cymodocea nodosa*	This study
**ATLANTIC WATERS**								
5 strains (Spain, Vigo)	**	**	**	**	**	OA = 5–24.5, DTX-1 = 6–14.3	**	Lee et al. (1989) [31]
PL100A (USA, Florida, Knight Key)	≈20–40 Opt = 26	≈20–40 Opt = 30	120–4400 µW·cm^−2^ Opt = 4000 (14L:10D)	K	0.3,0.47,0.62	**	*Heterosiphonia gibbesii*	Morton and Norris (1990) [92]
PL1V (Spain, Atlantic region)	**	**	**	**	**	Toxic (unique indication)	**	Faust (1991) [140]
** (Canada, Nova Scotia, Mahone Bay)	20	**	150 (16L:8D)	F/2	**	OA and DTX-1 = equal proportions = 25 ng·mL^−1^ of culture	Seawater	Marr et al. (1992) [32]
PL2V (Spain, Vigo)	20	35.5 ± 0.5	24 (12L:12D)	K	0.092	OA, DTX-1 = 10–15% Total toxin content = 4.35–7.67	**	Morlaix and Lassus (1992) [91]
PL100A (USA, Florida, Knight Key)	≈19–35 Opt = 27	≈20–43 Opt = 30	1500–5500 µW·cm^−2^ Opt = 4500 (14L:10D)	K	µmax≈0.3–0.56	**	*Heterosiphonia gibbesii*	Morton et al. (1992) [98]
** (Canada, Nova Scotia, Mahone Bay )	5,10,15,20,25	**	150 (16L:8D)	F/2	0.1–0.7	OA+DTX-1 = 1.4–8.0 OA:DTX-1 = 1.37 ± 0.23	Seawater	Jackson et al. (1993) [44]
** (USA, Florida, Dry Tortugas)	26	**	150 (16L:8D)	K	0.16–0.75	OA = 7.5–14.2 DTX-1 = trace concentrations	****	Tomas and Baden (1993) [93]
PL2V (Spain, Vigo)	20	**	40 (12L:12D)	F/2	**	OA = 14.3, DTX-1 = 2.7	**	Barbier et al. (1999) [35]
Isolate 712 (Spain, Vigo)	20	**	10 nmol photons·m^−2^·s^−1^ (12L:12D)	PES	**	**	**	Zhou and Fritz (1994) [141]
** (Canada, Nova Scotia, Mahone Bay)	18 ± 1	32	90 ± 5 (14L:10D)	L1	0.1–0.15	DTX-4 = 1.8–7.8, OA = 0.37–6.6, DTX-1 = 0.04–2.6, OA-D8 = 0.02–1.5 fmol cell^−1^	**	Pan et al. (1999) [94]
19 strains (Spain, Ria of Vigo and Pontevedra)	19 ± 1	**	60–70 (14L:10D)	K	0.06–0.14	OA = 0.19–12.87, OA ester = 0.77–17.51, DTX-1 = 0–12.45, DTX-2 = 0–1.14, DTX-2 ester = 0–1.60	Macroalgae	Bravo et al. (2001) [39]
20 strains (United Kingdom, Fleet lagoon)	15,17	**	70,90 (12L:12D; 16L:8D)	L-2	Strain 2.9a: 0.11	OA = 0.42–17.13, DTX-1 = 0.41–11.29; DTX-4, DTX-4+O and DTX-4+ CH2+2O detected	Seawater, seaweeds, eelgrass	Nascimento et al. (2005) [40]
** (Cuba, NW Havana city)	22 ± 1	**	Fluorescent lamp of 40 W (12L:12D)	K	**	Cultured cells: DTX-1 = 7.15 Natural cells: DTX-1 = 4.2	*Padina sp*	Delgado et al. (2005) [107]
IO66-01 (Portugal, Lisbon Bay)	19 ± 1	35	40 (14L:10D)	F/2-Si	0.49	Total OA = 8.8–41.0 and DTX-1 = 2.5–12.0 OA-D6, OA-D8, OA-D9 esters detected	Seawater	Vale et al. (2009) [96]
CCAP1136/11 (Spain, Ria de Vigo)	20	38	35 (16L: 8D)	F/2	0.11–0.22	OA = 0.10–1.25 (Day1–15), Maximum OA = 11.27 ± 3.30 (Day 34)	**	Varkitzi et al. (2010) [95]
Dn35EHU, Dn37EHU, Dn38EHU (Spain, S-E Bay of Biscay)	17-,22	30,35	60 (12L:12D)	F/2	**	Toxic to *Artemia franciscana* nauplii (mortality of 86.9% after 24 h)	Macroalgae Seawater	Laza-Martinez et al. (2011) [58]
**PACIFIC WATERS**								
** (Japan, Okinawa, Ishigaki Island)	25	**	4000–8000 lx (18L:6D)	S-PES	**	Ether and Butanol soluble fractions toxic to mice Hemolytic activity on mouse blood cells. No effects on killifish	*Turbinaria ornata* and *Amphiroa sp*	Nakajima et al. (1981) [53]
** (Tahiti Island)	25	**	4000–8000 lx (18L:6D)	S-ES-1	**	OA (= PLT2) = 40 mg·10^−10^·cells Toxic to mice (minimum lethal dose = 200 µg·kg^−1^)	**	Murakami et al. (1982) [30]
** (Japan, Okinawa)	**	**	**	**	**	OA = 26, DTX-1 = 13	**	Lee et al. (1989) [31]
OK-8510, OK-8603A, OK-8603B (Japan, Okinawa)	**	**	**	**	**	Toxic (unique indication) Toxic (unique indication)	**	Faust (1991) [140] Faust (1991) [140]
SP-8708A, SP-8708D (Saipan Island)								
17 clones (Australia, N/S/S-E Heron Island)	28	**	52 (16L:8D)	K	0.2–0.35	OA = 1.31–5.88 ≈ Methyl-okadaic acid = 4.0–12.0	Macroalgae (*Phaeophytes, Rhodophytes*)	Morton and Tindall (1995) [34]
** (New Zealand, Northland, Rangaunu Harbour )	18 ± 1	**	100 (14L:10D)	GP	**	OA = 6.3 ± 1 Toxic to *Artemia salina* (50 cells = 50% death response in 24 h, 200 cells = 50% death response in 20 h)	Sediments	Rhodes and Syhre (1995) [33]
** (Japan, Sanriku coast)	15,20,25	**	170 (14L:10D)	T1	>0.2	OA = 0.3 to 1.3	*Sargassum confusum* *Carpopeltis flabellate*	Koike et al. (1998) [142]
P6 (New Caledonia)	25–29	30–34	50–90 (12L:12D)	F_10k_	0.27	OA = 1.1–15, 7-deoxy-okadaic acid = 0.2–1.5 Inhibition of PP2A activity	**	Holmes et al. (2001) [90]
PL01 (Taiwan)	25	**	** (14L:10D)	K –ES	**	4-hydroxyprorocentrolide, 14-O-acetyl-4-hydroxyprorocentrolide	Seaweeds	Lu et al. (2001) [110]
PRL-1 (Mexico, Gulf of California, El Pardito)	22 ± 1	**	4 × 20 W fluorescent lamps (12L:12D)	ES-SI	0.107	OA, DTX-1, (OA:DTX1) = (1:2) OA+DTX-1 = 5.2 (HPLC-MS) Total toxin content = 19 (mouse bioassay) Toxic to mice, to *Artemia franciscana* larvae and to the yeast *Debaryomyces hansenii*	Rocky substrate	Heredia-Tapia et al. (2002) [48]
PL021117001 (Taiwan, Northern coast)	25	**	** (16L:8D)	K-ES	**	Prorocentin, OA Inhibitory activity of Prorocentin against human colon adenocarcinoma and human malignant melanoma	**	Lu et al. (2005) [42]
** (Southern China, Hainan Island, Coast of Sanya)	25	**	70 (12L:12D)	K	**	OA, two diol esters (OA-D10a and OA-D10b)	Macrophytes	Li et al. (2012) [111]
**INDIAN WATERS**								
8 strains (La Reunion, Mayotte, Europa, and Mauritius Islands)	26	**	90 (12L:12D)	PPES	**	OA = 128.3–6261.3 ng·mg^−1^ crude extract Inhibitory effect on PP2A Cytotoxic activity on FR3T3 fibroblasts	**	*Bouaicha* et al. (2001) [36]
**INDO- PACIFIC WATERS**								
3 strains (Peninsula and East of Malaysia)	26–27	32	30 (12L:12D)	ES-DK	**	High toxicity to *Artemia franciscana* larvae	*Sargassum spp*. *Padina spp.* Dead corals	Mohammad Noor et al. (2007) [101]

** No Data; ≈ Seen in figures. Cellular toxin content is expressed in pg·cell^−1^ or other specified unit. Irradiance is expressed in µmol photons·m^−2^·s^−1^ orother specified unit. Abbreviations: Opt = Optimum value; PP2A = protein phosphatase type 2A; PES = Provasoli’s ES medium; S-PES Seawater with Provasoli's ES supplement; S-ES = Seawater with ES-1supplement; K-ES = K nutrient enriched Seawater; PPES = Provasoli-Pintner modifed ES natural seawater.

**Table A3:** Summary for *Coolia monotis* established laboratory cultures from various marine ecosystems. Culture conditions, growth rates and toxicity are specified when available.

Strain and Sampling Location	Temperature (°C)	Salinity	Irradiance (µmol photons·m^−2^·s^−1^) and L:D Cycle (h)	Culture Medium	Growth Rate (d^−1^)	Toxicity: Detected Compounds, Hemolytic Activity, Toxicity to Mice and Other Organisms	Isolated from	Reference
**MEDITERRANEAN SEA**								
CNR-CMA4 (Italy, Ionian Sea, Taranto)	17 ± 1	**	100 (14L:10D)	K, F/20, F/2	**	**	Seawater *Rhodophyceae Phaeophyceae*	Penna et al. (2005) **^a^** [20] (MI)
CNR-CMB2 (Italy, Tyrrhenian Sea, Ganzirri)	-	-	-	-	-	-	*-*	-
IEO-CM6V (Spain, Almeria)	-	-	-	-	-	-	*-*	-
SZN-CM43 (Italy, Tyrrhenian Sea, Napoli)	-	-	-	-	-	-	*-*	-
** (Greece, North Aegean Sea, Thermaikos Gulf)	23	≈33	60–70 (14L:10D)	L1	**	**	Seawater	Dolapsakis et al. (2006) **^a^** [143] (MI)
** (Greece, North Aegean coasts)	19 ± 1	**	70 (14L:10D)	F/2, K	**	**	Macrophytes	Aligizaki and Nikolaidis (2006) **^a^** [77]
** (Italy, Northern Ionian Sea, Mar Piccolo, Mar Grande and Lido Bruno)	24 ± 2	37	100 (12L:12D)	F/2	**	Live cells: no effects on *P. lividus* embryonic development Cell lysate: inhibition of *P. lividus* embryos development, low hemolytic activity on human erythrocytes, not toxic to *Artemia salina* nauplii	Seawater Rocks scraping	Pagliara and Caroppo (2012) **^b^** [54]
CMBZT14 (Tunisia, Bizerte Bay)	25	36	80 (12L:12D)	ENSW	0.35	Unknown molecule: Chromatographic peak at 5.6 min with a mass *m/z* = 1061.768	*Cymodocea nodosa*	This study **^a^** (MI)
**ATLANTIC WATERS**								
Clones 542, 543 (Carribean Sea, Leeward Islands, IIe St. Barthelemy, Port de Gustavia)	28	35	300 ft-c (12L:12D)	GPM	**	Mouse bioassay: lack of water or lipid soluble ciguatera toxins in *C. monotis* extracts	Tide-pool	Besada et al. (1982) **^a^** [137]
** (Central America, Twin Cays, Belize)	23 ± 0.5	36	30–90 (12L:12D)	Erdschreiber	Dt = 3–4 days	**	Floating detritus Surface sediment	Faust (1992) **^a^** [97]
CM300A (USA, Florida, Knight Key)	≈21–35, Opt = 29 °C	≈23–43, Opt = 33	1500–5500, Opt = 5300 µW·cm^−2^ (14L:10D)	K	µmax ≈ 0.2–0.6	**	*Heterosiphonia gibbesii*	Morton et al. (1992) **^c^** [98]
CCMP304 (Spain, Ria de Vigo)	5,10,15,20,25,30,35	18–37	100 (14L:10D)	GP	**	Not toxic to mice	Macroalgae Sediments	Rhodes et al. (2000) **^a^** [55]
IEO-CM2V (Spain, Vigo) CCMP1345 (USA, Florida)	17 ± 1	**	100 (14L:10D)	K, F/20, F/2	**	No hemolytic activity on human erythrocytes	Seawater *Rhodophyceae Phaeophyceae*	Penna et al. (2005) **^a^** [20] (MI)
IEO-CM3V (Spain, Vigo) NICMM-RIKZ3, NICMM-RIKZ4 (North Sea, Netherlands, Yerseke)	17 ± 1	**	100 (14L:10D)	K, F/20, F/2	**	**	Seawater *Rhodophyceae Phaeophyceae*	Penna et al. (2005) **^a^** [20] (MI)
Dn23EHU,Dn24EHU,Dn25EHU,Dn26EHU (Spain, S-E Bay of Biscay)	17–22	30–35	60 (12L:12D)	F/2	**	Not toxic to *Artemia franciscana* nauplii	Seawater Macroalgae	Laza-Martinez et al. (2011) **^a^** [58] (MI)
32 strains (Atlantic coast of the Iberian Peninsula)	20	**	80 (12L:12D)	F/2	**	**	Seawater Macroalgae	David et al. (2014) **^a^** [144] (MI)
**PACIFIC WATERS**								
** (Japan, Okinawa, Ishigaki Island)	25	**	4000–8000 lx (18L:6D)	S-PES	**	Not toxic to mice Hemolytic activity on mouse blood cells No effects on killifish	*Turbinaria ornata* *Amphiroa sp*	Nakajima et al. (1981) **^a^** [53]
** (Japan, Okinawa, Coast of Motobu)	23–28	**	1500–3000 Lx (18L:6D)	S-ES-1	**	Ceramide with a 2-hydroxy-15-methyl-3-octadecenoyl moiety	****	Tanaka et al. (1998) **^a^** [116]
** (Australia, Queensland, Platypus Bay)	25	**	50–60 (12L:12D)	F_10K_	**	Cooliatoxin: a monosulfated polyether toxin (*m/z* = 1061.5) Butanol soluble fraction lethal to mice (LD50 = 1 mg·kg^−1^ in mice)	*Cladophora sp*	Holmes et al. (1995) **^d^** [52]
CAWD39 (New Zealand, Northland, Ninety Mile Beach)	20,25/18 Opt = 25	15–43 Opt>28	100 (14L:10D)	GP	25 °C:Dt = 4 days	Toxic to larvae of *Artemia salina* and *Haliotis virginea*	*Foliose red Landsburgia quercifolia*	Rhodes and Thomas (1997) **^c^** [56]
CAWD39 (New Zealand, Northland, Ninety Mile Beach)	5,10,15,20,25,30,35	18–37	100 (14L:10D)	GP	**	Not Toxic to mice Two analogs of unknown polyether compounds detected	Macroalgae Sediments	Rhodes et al. (2000) **^c^** [55]
CAWD77 (New Zealand, Northland, Rangiputa)	5,10,15,20,25,30,35	18–37	100 (14L:10D)	GP	**	Toxic to mice Cytotoxic	Macroalgae Sediments	Rhodes et al. (2000) **^c^** [55]
CMLHT01 (South China Sea, Hainan island)	**	**	**	**	**	Cooliatin = dioxocyclononane (C15H22O5)	Seaweeds	Liang et al. (2009) **^a^** [117]
CAWD151 (Cook Islands, Rarotongan lagoons)	25	**	80 (14L:10D)	F/2	**	Low toxicity to mice	*Halimeda sp.*	Rhodes et al. (2010) **^c^** [114] (MI)
** (Coast of Vietnam)	26	32	25 (12L:12D)	T	**	**	Macroalgae Seagrasses	Ho and Nguyen (2014) **^a^** [145]

** No Data; ≈ Seen in figures; (MI) = Molecular identification performed; *^a^ C. monotis*; *^b^ C.* cfr. *monotis*; *^c^* Firstly described as *C. monotis* then reclassified as *C. malayensis, ^d^* Firstly described as *C. monotis* then re-identified as *C. tropicalis.* Irradiance is expressed in µmol photons.m^−2^·s^−1^ orother specified unit.
